# Cross-tissue integrative transcriptomic and multimodal analyses suggest shared immune signatures linking lupus nephritis and cutaneous lupus erythematosus

**DOI:** 10.3389/fimmu.2026.1716516

**Published:** 2026-02-24

**Authors:** Meilu Li, Changze Song, Zilong Wang, Huisheng Yuan, Zhiqiang Zhang, Yu Sun, Fu Zhang, Songjuan Wang, Hongcheng Sun, Hanshu Zhao, Linyu Zhu, Di Wang, Yuzhen Li

**Affiliations:** 1 Department of Dermatology, The Second Affiliated Hospital of Harbin Medical University, Harbin, China; 2 Department of Dermatology, The Seventh Affiliated Hospital, Sun Yat-Sen University, Shenzhen, China; 3 Department of Andrology, The Seventh Affiliated Hospital, Sun Yat-Sen University, Shenzhen, China; 4 Department of Burns and Plastic Surgery, The Seventh Affiliated Hospital, Sun Yat-Sen University, Shenzhen, China; 5 Digestive Diseases Center, The Seventh Affiliated Hospital, Sun Yat-sen University, Shenzhen, China; 6 Department of Orthopaedic Surgery, The Seventh Affiliated Hospital, Sun Yat-sen University, Shenzhen, China; 7 Department of Medical Ultrasonic, The Seventh Affiliated Hospital, Sun Yat-sen University, Shenzhen, China; 8 Department of Gastroenterology, The First Affiliated Hospital of Harbin Medical University, Harbin, China; 9 Department of Neurology, The First Affiliated Hospital of Harbin Medical University, Harbin, China; 10 Health Management Center, The Seventh Affiliated Hospital, Sun Yat-sen University, Shenzhen, China

**Keywords:** cutaneous lupus erythematosus, lupus nephritis, machine learning, shared immunopathological mechanisms, single-cell RNA sequencing, systemic lupus erythematosus, weighted gene co-expression network analysis

## Abstract

**Background:**

Lupus nephritis (LN) and cutaneous lupus erythematosus (CLE) are major organ manifestations of systemic lupus erythematosus (SLE), imposing significant health and economic burdens due to their chronic course. This study aims to explore putative shared molecular signatures and hypothesis-generating therapeutic targets by examining the expression profiles of genes associated with LN and CLE.

**Methods:**

We analyzed gene expression profiles from LN and CLE using bulk transcriptome analysis, single-cell RNA sequencing, and machine learning approaches. Differentially expressed genes (DEGs) were identified, and weighted gene co-expression network analysis (WGCNA) was employed to reveal gene modules associated with clinical traits. Functional enrichment analyses were performed to characterize implicated pathways. Machine learning algorithms, including LASSO, SVM-RFE, and random forest, were applied to screen for putative biomarkers. Single-cell datasets were used to determine the cellular distribution of candidate genes, and validation was conducted in the lupus mouse model C57BL/6-FasLpr.

**Results:**

A total of 361 DEGs in LN and 711 DEGs in CLE were identified, with 99 overlapping genes. We combined overlapping genes from WGCNA and DEGs to conducted enrichment analysis, highlighted disease-associated mechanism enriched in immune-related pathways, particularly type I interferon signaling. Machine learning analysis identified six hub genes, PDE4B, ISG20, IFI27, PARP12, IFI44 and GATA3, most of which demonstrated diagnostic value with AUC values >0.7. Single-cell RNA sequencing confirmed their expression in T cells, B cells, and NK cells, implicating them in immune dysregulation. *In vivo* validation in Lpr mice revealed elevated serum IFN-β levels and decreased ratio of CD4/CD8, consistent with human transcriptomic findings.

**Conclusion:**

This integrative analysis establishes a shared molecular signature between LN and CLE. The identified hub genes represent promising hypothesis-generating molecular signatures and hypothesis-generating therapeutic targets, with potential to improve risk stratification, guide early intervention, and support precision medicine approaches for lupus comorbidities.

## 1 Introduction

Lupus nephritis (LN) and cutaneous lupus erythematosus (CLE) are autoimmune disorders. They pose significant challenges because of their complex pathophysiological mechanisms and their profound impact on patients’ health and quality of life. LN, a severe form of systemic lupus erythematosus, affects around 40-50% of patients and may progress to chronic kidney disease, increasing morbidity (1). Conversely, CLE presents with various skin symptoms. It may occur alone or alongside systemic disease, making diagnosis and treatment more difficult (2). Moreover, both conditions cause significant economic burdens due to their chronic management and treatment needs.

Currently, treatment approaches for LN and CLE mainly involve immunosuppressants and corticosteroids. However, these interventions have significant drawbacks, such as adverse effects and variable efficacy in some patient populations (1). This predicament underscores the need to explore novel therapeutic pathways and to generate hypotheses regarding molecular signatures that may assist early disease stratification and guide individualized management. Recent studies emphasize the need for innovative approaches to meet the clinical challenges of these disorders, especially regarding renal involvement in SLE patients with skin manifestations (3).

The pathogenesis underlying LN and CLE are intricate. Accumulating evidence suggests that genetic and molecular elements play critical roles in their development. Prior studies have found changes in immune regulation and gene expression that may drive disease progression (4). However, we still lack a clear understanding of specific gene expression patterns and how they relate to disease mechanisms, particularly regarding clinical phenotypes and treatment responses. Addressing this gap in knowledge is vital for advancing our understanding of LN and CLE, ultimately improving patient outcomes.

In this study, we employ an integrative strategy. This strategy combines bulk transcriptome analysis, single-cell RNA sequencing, and machine learning methodologies to explore gene expression profiles associated with LN and CLE. This comprehensive analysis facilitates the identification of putative biomarkers and therapeutic targets, providing deeper insight into the underlying molecular mechanisms. The incorporation of machine learning is particularly noteworthy, as it assists in identifying candidate genes that predict clinical outcomes, thereby enhancing diagnostic precision and informing treatment strategies.

Despite growing transcriptomic and single-cell studies characterizing lupus nephritis and cutaneous lupus erythematosus individually, several critical gaps remain. Most existing studies focus on single organs or single data modalities, leaving it unclear whether shared molecular signatures and regulatory networks exist across renal and cutaneous tissues that could explain the frequent co-occurrence and systemic nature of lupus. In particular, the identification of cross-tissue biomarkers that link immune dysregulation in the skin to renal involvement remains poorly defined. To address these gaps, an integrative analytical framework is required. Bulk transcriptomic analysis enables the detection of conserved gene expression patterns and co-expression modules across tissues, while single-cell RNA sequencing provides cellular resolution to contextualize these signals within specific immune cell populations. Moreover, machine learning–based approaches allow for unbiased feature selection and prioritization of biomarkers with potential diagnostic and translational relevance. Therefore, in this study, we integrated bulk transcriptomics, weighted gene co-expression network analysis, single-cell RNA sequencing, and machine learning to systematically identify shared immune signatures, key regulatory genes, and putative biomarkers underlying the comorbid pathogenesis of LN and CLE.

The primary objective of this study is to identify hypothesis-generating molecular signatures that may signal renal involvement in patients with SLE who initially present with cutaneous manifestations. Rather than reiterating the well-established role of type I interferon in lupus, this work seeks to advance current knowledge by integrating bulk transcriptomic data from glomerular and cutaneous biopsies with single-cell RNA sequencing and machine-learning–based feature prioritization, thereby enabling a unified analysis across distinct tissue compartments. Through this multimodal analytical strategy, we aim to delineate a convergent molecular axis involving cAMP-dependent inflammatory signaling and interferon-stimulated immune activation, which may mechanistically link cutaneous inflammation to renal injury. These findings are intended to serve as translational hypotheses, providing a framework for future mechanistic and clinical studies rather than definitive diagnostic signatures, and to inform subsequent efforts toward biomarker-guided patient stratification and targeted therapeutic development in lupus.

## 2 Materials and methods

### 2.1 Acquisition and pre-processing of bulk transcriptome data

We retrieved three microarray gene expression datasets (LN-GSE32591, CLE-GSE95474, LN-GSE112943 and CLE-GSE112943) along with two single-cell datasets (CLE-GSE294296 and LN-GSE135779) from the Gene Expression Omnibus (GEO) repository, selected based on their relevance to LN or CLE (**Table 1**) (5). The GSE32591 dataset included 32 LN patients and 15 healthy controls (**Supplementary Table 1**). GSE95474 encompassed 5 CLE patients and 5 controls (**Supplementary Table 2**). GSE112943 consisted of 14 LN patients, 16 CLE patients and 7 healthy renal samples, 10 healthy skin samples. GSE294296 contained 4 CLE patients and 3 healthy controls. GSE135779 consisted of 7 SLE patients and 5 healthy controls. The raw gene expression datasets were acquired from the GEO database via the GEOquery package. After obtaining the expression matrix, probe IDs were converted to gene symbols. Probes without annotations were discarded, and duplicated gene symbols were addressed by averaging their expression values to maintain data consistency. Relevant clinical and experimental information was extracted from the sample metadata. To assess the necessity for normalization, we calculated the maximum expression value. If this value surpassed a predetermined threshold, a transformation was applied to mitigate the impact of outliers and normalize the data distribution. The final expression matrix, along with the corresponding metadata, was prepared for subsequent analyses.

**Table 1:** Information of GEO datasets used in this study.

Database	Platform	Experiment Type	Disease	Sample type	Control (n)	Case (n)	Total (n)
GSE32591	GPL14663	Microarray	LN	kidney	14	32	46
GSE95474	GPL16699	Microarray	CLE	skin	5	5	10
GSE112943	GPL10558	Microarray	LN	kidney	7	14	21
GSE112943	GPL10558	Microarray	CLE	skin	10	10	20
GSE294296	GPL24676	Single-cell	CLE	skin	3	5	8
GSE135779	GPL20301	Single-cell	SLE	PBMC	5	7	12

### 2.2 Identification and visualization of differentially expressed genes

Utilizing the limma R package, we processed and analyzed the GSE32591 and GSE95474 datasets, adhering to a standardized workflow to ensure data comparability. We established rigorous criteria for the identification of differentially expressed genes (DEGs), necessitating a corrected p-value of less than 0.05 and |log_2_ fold change| greater than 1. To illustrate the expression variations of the DEGs, we employed pheatmap and ggplot2 advanced visualization capabilities.

### 2.3 Weighted gene co-expression network analysis

A weighted gene co-expression network analysis (WGCNA) was performed on the GSE32591 and GSE95474 datasets to investigate gene co-expression patterns associated with clinical traits. The expression data underwent preprocessing, wherein genes exhibiting missing values or demonstrating zero variance were eliminated. To minimize noise, genes with low variability (standard deviation ≤ 0.5) were also filtered out (6). The optimal soft-thresholding power was determined automatically through the pickSoftThreshold function within the WGCNA package, ensuring that the resulting network adhered to the scale-free topology criterion (scale-free R² ≥ 0.9). Utilizing the selected power, an adjacency matrix was generated and subsequently transformed into a topological overlap matrix (TOM) to assess network connectivity. Hierarchical clustering based on TOM dissimilarity was conducted to identify gene modules. Modules were identified utilizing a dynamic tree cut method, with a minimum module size of 20 genes, a sensitivity parameter (deepSplit) of 2, and a module merging threshold (mergeCutHeight) set at 0.25. Genes that could not be confidently allocated to any module were categorized into the grey module. Ultimately, module eigengenes were computed, and Pearson correlation analyses were executed between the module eigengenes and clinical traits to pinpoint modules associated with specific traits (7).

### 2.4 Enrichment analysis by using GO and KEGG

Comprehensive functional enrichment analyses were conducted to elucidate the potential functions of the identified targets. Gene Ontology (GO) and the Kyoto Encyclopedia of Genes and Genomes (KEGG) represent two distinct gene function annotation databases frequently employed for the biological interpretation of high-throughput data. The GO database organizes gene functions into three principal domains: molecular function (MF), cellular component (CC), and biological process (BP), facilitating a thorough classification of gene attributes. Conversely, KEGG serves as an integrative resource that emphasizes metabolic and signaling pathways, cataloging gene sets while also illustrating the intricate interactions among genes, proteins, and metabolites. In this investigation, both enrichment analyses were executed to thoroughly examine the biological pathways enriched by the shared differentially expressed genes (DEGs) identified between SLE and UC. The enrichment analysis was performed utilizing the R package clusterProfiler (8), with GO annotations sourced from the GO database (9) and pathway data from KEGG (10). For the GO enrichment assessment, the parameter showNum=10 was applied to extract the top eight terms across the three GO categories (MF, CC, BP). Similarly, for KEGG, the showNum=10 parameter was employed to present the top ten enriched pathways. The visualization of enrichment outcomes was accomplished through the ggplot2 package in R. Functional terms exhibiting P < 0.05 were regarded as statistically significant and classified as enriched terms.

### 2.5 Gene set enrichment analysis

Gene set enrichment analysis (GSEA) was executed using the software developed by the Broad Institute to identify significantly enriched biological pathways between disease and control groups. Genes were ranked according to the signal-to-noise metric, and enrichment scores (ES) were computed to ascertain whether members of predefined gene sets were overrepresented at either the upper or lower ends of the ranked list. Statistical significance was evaluated through 1,000 phenotype permutations, with normalized enrichment scores (NES) calculated to adjust for potential biases. To ensure robustness and interpretability, only those gene sets containing between 15 and 500 genes were included.

### 2.6 PPI networks construction

The analysis of protein–protein interactions (PPI) offers essential insights into the functional interrelations among proteins and their collaborative biological functions (11). This study constructed PPI networks to investigate the interactions among the overlapping genes identified in SLE and UC. The STRING database (https://string-db.org/) was employed to create a comprehensive interaction network (7). A minimum confidence score threshold of 0.4 was established to ensure the dependability of the predicted interactions. The resultant PPI networks were visualized utilizing Cytoscape software (version 3.8.1; http://cytoscape.org/), a widely recognized platform for the visualization and analysis of networks. Cytoscape accommodates a diverse range of biological network types, including PPI networks, transcriptional regulatory circuits, miRNA-mRNA interaction networks, competing endogenous RNA (ceRNA) networks, and diagrams illustrating pathway crosstalk, rendering it a powerful and adaptable tool for network-based biological interpretation.

### 2.7 Disease ontology enrichment analysis

The Disease Ontology (DO) database is a standardized ontology resource that integrates disease-related information across multiple biomedical databases. Specifically, DO enrichment was conducted using the clusterProfiler (v4.0+) R package, which applies a hypergeometric test to evaluate whether the input gene set is significantly enriched in specific disease categories within the DO database.

### 2.8 Single-cell transcriptome data

We exclusively downloaded the adult CLE dataset from GSE294296 and SLE dataset from GSE135779. The preprocessing of single-cell transcriptome data adhered to previously described methodologies, employing the Seurat package (7). In summary, quality control filtering retained cells with mitochondrial gene content (percent.MT) below 10%, total RNA counts (nCount_RNA) exceeding 1000, detected gene features (nFeature_RNA) surpassing 200, and a hemoglobin gene expression proportion (percent.HB) of less than 0.05. Following normalization, the top 2000 highly variable genes across all cells, excluding mitochondrial and hemoglobin genes, were selected for subsequent analysis. Data scaling was executed using the ScaleData function, and dimensionality reduction was conducted via principal component analysis (RunPCA). Cell clustering was accomplished through the utilization of FindNeighbors and FindClusters, with visualization performed using RunUMAP. Cell type annotation was facilitated by marker genes identified for each cluster, utilizing the SingleR package along with manual curation. Lastly, expression patterns of selected genes were visualized employing FeaturePlot and VlnPlot.

### 2.9 Machine learning selection of putative biomarkers

Machine learning models were developed to classify samples based on the expression levels of 6 selected genes: PDE4B, ISG20, IFI27, PARP12, IFI44, GATA3. The corresponding sample labels served as the outcome variable. The dataset was randomly partitioned into training and validation sets at a ratio of seventy to thirty percent, ensuring stratification by class labels to maintain the original class distribution. Feature values were standardized using the StandardScaler method to achieve a mean of zero and unit variance. Three classification algorithms were utilized: **LASSO** regression was implemented using the glmnet package, with gene expression values as predictors and disease status as the response variable. Feature selection was achieved by imposing an L1 penalty to shrink regression coefficients, and the optimal regularization parameter (λ) was determined through 10-fold cross-validation based on the minimum mean cross-validated error. **Random Forest** analysis was conducted using the randomForest package to construct an ensemble of decision trees and rank feature importance based on the mean decrease in Gini impurity. Key hyperparameters, including the number of trees (ntree) and the number of variables randomly sampled at each split (mtry), were optimized through cross-validation to minimize classification error, and genes with consistently high importance scores were retained as candidate features. In parallel, **support vector machine (SVM)** analysis was performed using the e1071 package with a radial basis function (RBF) kernel. Feature selection was achieved via recursive feature elimination, which iteratively removed features with the lowest weights. Hyperparameters, including the penalty parameter (C) and kernel width (γ), were optimized using grid search combined with cross-validation. Each model was trained on the training set and subsequently evaluated on the validation set. The performance of the models was measured using receiver operating characteristic curves and the area under the curve metric. ROC curves were plotted for each model independently. The LASSO logistic regression model’s regularization path was illustrated by plotting the coefficients across various regularization parameters. To determine the optimal regularization strength, five-fold cross-validation was employed, and the average cross-validation accuracy along with its standard deviation was graphed against the regularization parameters. All developed models were preserved to ensure reproducibility. The significance of features or coefficient weights was extracted from each model and ranked to evaluate the contribution of each gene to the classification process.

### 2.10 Animal model and flow cytometry

C57BL/6-FasLpr (Lpr) and C57BL/6 (B6) mice were purchased from Jackson Laboratory, and housed in specific pathogen-free conditions in the animal facility of Topbiotech Co., Ltd in Shenzhen, China. All the experiments were approved by the Institutional Animal Care and the Ethics Committee of Topbiotech Co., Ltd (Shenzhen).

Single-cell suspension come from peripheral lymph nodes and peripheral blood mononuclear cells (PBMCs). For analysis of surface markers, cells were stained in PBS containing 1% (w/v) BSA on ice for 30 min, with Super Bright 600–labeled anti-CD4 (Clone: SK-3; eBioscience), BV510-labeld anti-CD8a (clone:53-6.7; BioLegend); APC-Cy7-labeled TCRβ (clone: H57-597; BioLegend), BV605-labeled B220 (clone: RA3-6B2; BioLegend),. Flow cytometry was performed on the Beckman Cytoflex s, and analysis was performed using FlowJo software.

### 2.11 Immune infiltration analysis

Immune cell infiltration in kidney and skin biopsy datasets was estimated using the CIBERSORT algorithm with the LM22 signature matrix, which quantifies the relative proportions of 22 immune cell subsets. Normalized expression matrices were analyzed with 1,000 permutations, and samples with a CIBERSORT P value < 0.05 were retained for further analysis. Differences in immune cell proportions between disease and control groups were assessed using the Wilcoxon rank-sum test. Spearman correlation analysis was performed to evaluate associations between immune cell fractions and key hub genes, and the results were visualized using the “ggplot2” package.

### 2.12 Statistical analysis

All statistical analyses were conducted utilizing R (version 4.3.1), R Studio (version 1.0.143), Python (version 3.11) and GraphPad Prism (version 10). Results are presented as mean ± standard deviation. The parametric Student’s t-test or the nonparametric Mann–Whitney test was employed for comparisons between two groups. For comparisons involving more than two groups, a parametric one-way analysis of variance (ANOVA) was executed, followed by the Bonferroni method to determine significance. p-value of less than 0.05 was regarded as statistically significant.

## 3 Results

### 3.1 Data preprocessing and identification of candidate genes in lupus nephritis and cutaneous lupus erythematosus

The study design is illustrated in **Supplementary Figure 1**. Gene expression data were sourced from skin samples of cutaneous lupus erythematosus (CLE) and kidneys biopsies of lupus nephritis (LN). For LN, samples from 32 patients alongside 15 healthy controls were acquired from the GEO datasets (GSE32591). In the case of CLE, the analysis included 5 patient samples and 5 healthy control samples sourced from GSE95474.

**Figure 1:**
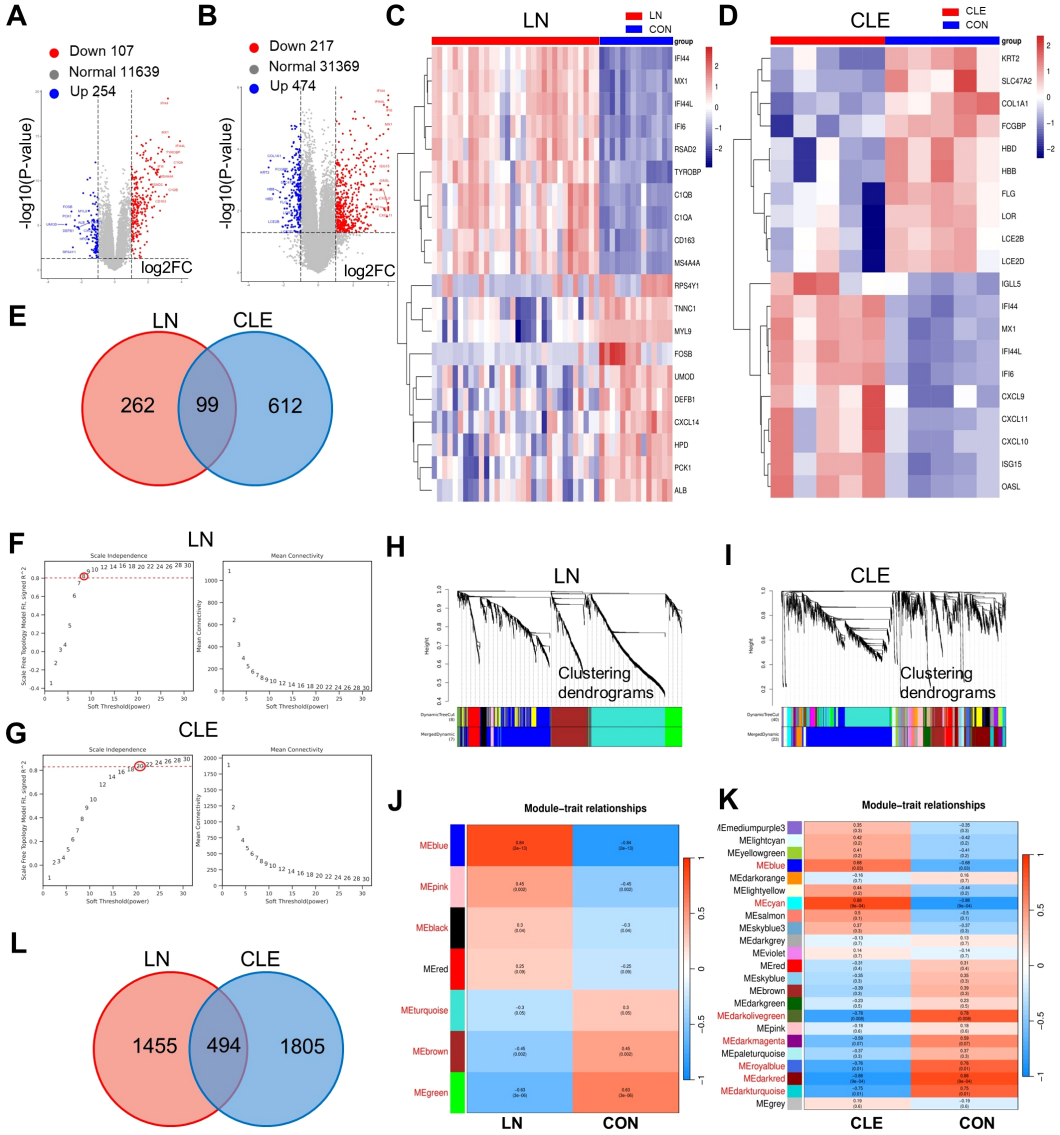
Identification of DEGs and construction of WCGNA in LN and CLE. **(A, B)** Volcano plot showing the distribution of DEGs in LN **(A)** and CLE **(B)**. Blue dot represents down-regulated genes and orange dot represents up-regulated genes. **(C, D)** Heatmap of genes with the prominent differential expression in the LN **(C)** and CLE datasets. **(E)** Overlay of DEGs of LN and CLE. **(F, G)** Mean connectivity for scale independence and soft threshold (β) in the LN **(F)** and CLE **(G)** cohort. **(H, I)** Clustering dendrograms of genes in LN **(H)** and CLE **(I)**. **(J, K)** Heatmap of the correlation analysis of module eigengenes with clinical traits between LN **(J)** or CLE **(K)** and control (CON). Red color and blue color represent positive and negative correlation, respectively. Red-annotated modules indicate |r| > 0.3. **(L)** Venn diagram illustrating the overlapping genes among the most strongly correlated modules (|r|>0.3, p<0.05) in LN and CLE.

The differentially expressed genes (DEGs) were depicted using volcano plots and heatmaps. The heatmaps clearly illustrated a distinction between the disease and healthy control groups, thereby affirming the reliability and biological significance of the differential expression findings. A total of 12,000 genes were analyzed for differential expression in the LN group, among which 254 were significantly upregulated and 107 were downregulated (**Figure 1A**). In the CLE group, differential expression analysis identified 474 upregulated and 217 downregulated genes out of 32,060 analyzed genes (**Figure 1B**). The corresponding heatmaps display representative upregulated and downregulated genes with notable expression changes in these 2 groups (**Figures 1C, D**). Ultimately, 361 DEGs were identified within the LN cohort and 711 within the UC cohort. A total of 99 overlapping DEGs were identified across the two disease datasets (**Figure 1E**). Comprehensive details regarding these overlapping genes can be found in **Supplementary Table 3**.

To investigate the potential correlations between gene expression profiles and clinical phenotypes in LN and CLE, we undertook weighted gene co-expression network analysis (WGCNA). Following the exclusion of low-variance genes to mitigate noise, the optimal soft-thresholding power (β) was established in accordance with scale-free topology criteria, yielding β = 8 (R² > 0.84) for the LN dataset and β = 20 (R² > 0.81) for the CLE dataset (**Figures 1F, G**). Utilizing these thresholds, WGCNA was performed, and modules were delineated through hierarchical clustering using a dynamic tree cut algorithm. Modules characterized by high similarity were amalgamated, resulting in the identification of a total of ten distinct modules. The LN dataset contained 7 modules, while the CLE dataset comprised 23 modules (**Figures 1H, I**). To pinpoint modules pertinent to the disease, we focused on those exhibiting the most pronounced positive or negative correlations with clinical characteristics (|r| > 0.3). In the LN dataset, the blue (r =0.84), pink (r = 0.45), black (r =0.3), turquoise (r =-0.3), brown (r =-0.45), and green (r =-0.63), modules displayed significant associations with disease status (**Figure 1J**). For the CLE dataset, the blue (r =0.68), cyan (r =0.88), dark olivegreen (r =-0.78), darkmagenta (r = -0.59), and royalblue (r = –0.76), darkred (r =-0.88), and darkturquoise (r =-0.75) modules exhibited the strongest correlations (**Figure 1K**). The corresponding scatter plots illustrating the relationship between modules and traits can be found in **Supplementary Figure 2**. Following this, we amalgamated all positively and negatively correlated modules from both diseases to uncover shared molecular signatures, which resulted in the identification of 494 overlapping genes potentially involved in the pathogenesis of both LN and CLE (**Figure 1L**). The comprehensive list of these candidate genes is available in **Supplementary Table 3**.

### 3.2 Functional enrichment analysis

**Figure 2:**
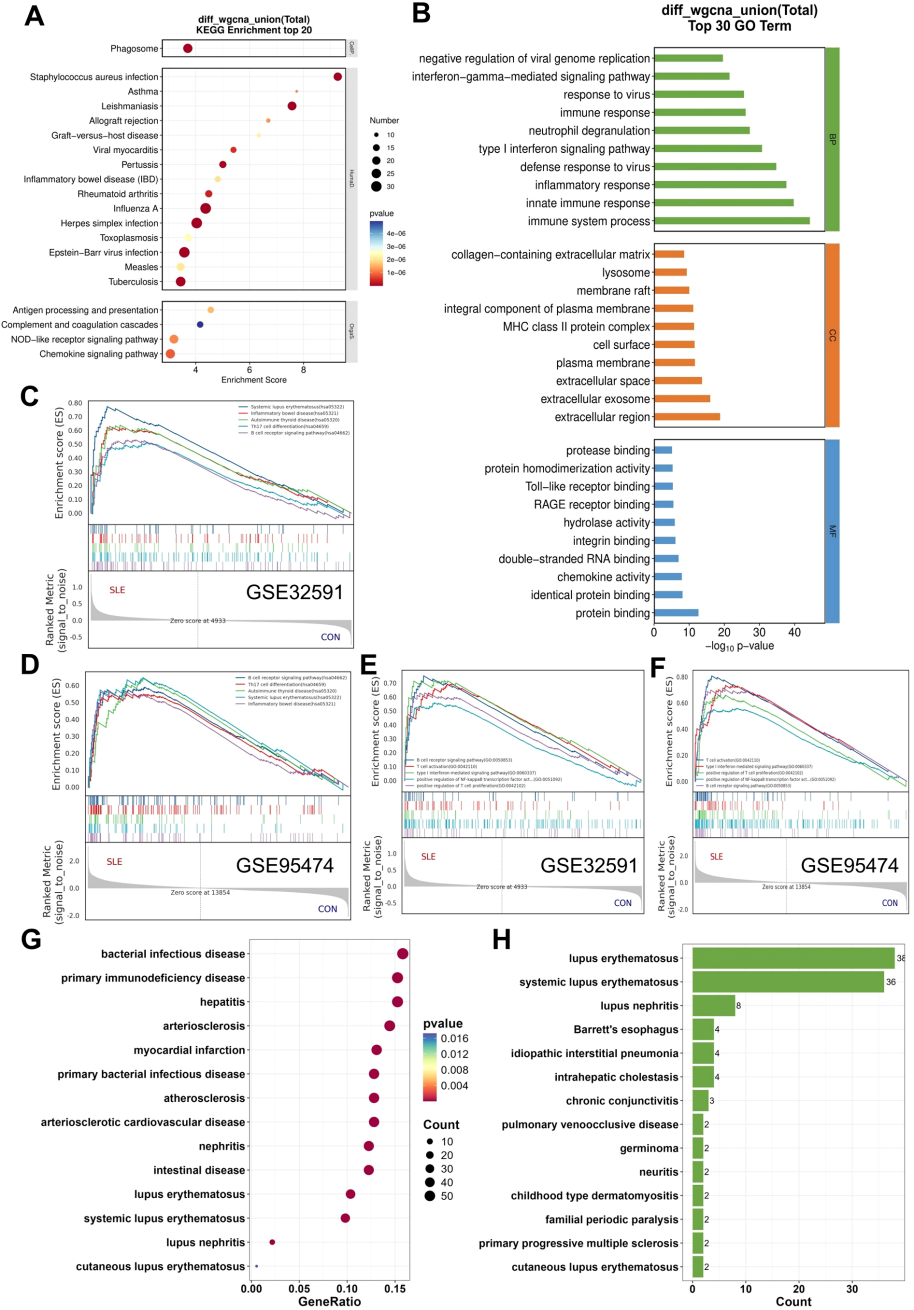
Functional enrichment analysis. **(A)** KEGG pathway enrichment analysis of the overlapping genes. **(B)** GO enrichment analysis of the overlapping genes. **(C, D)** Gene Set Enrichment Analysis (GSEA) based on the KEGG database, showing the top five upregulated biological processes in LN-GSE32591 **(C)** and CLE-GSE95474 **(D)** datasets. **(E, F)** GSEA based on the GO database, showing the top five upregulated biological processes in LN-GSE32591 **(E)** and CLE-GSE95474 **(F)** datasets. **(G)** DO enrichment analysis of the union gene set. **(H)** DisGeNET enrichment analysis of the union gene set.

In the functional enrichment analysis, 99 overlapping genes were identified between the LN and CLE modules, while 494 genes were shared among the differentially expressed genes (DEGs). It is important to note that the modules derived from WGCNA encompass a collection of genes with similar expression patterns, which may not necessarily represent the entire spectrum of DEGs or could even differ significantly from DEGs that are critical for disease advancement. To investigate the common pathogenesis of LN and CLE, we conducted functional enrichment analyses on the 497 candidate genes obtained from WGCNA and DEGs combined overlapping genes (**Supplementary Table 3**.). The KEGG and GO enrichment analysis suggested both LN and CLE patients were involved in immune-related pathways (**Figures 2A, B**, **Supplementary Figures 3A-D**). In the Biological Process (BP) category, the most prominent terms included immune system process, innate immune response, and type I interferon signaling pathway, which reflect the activation of the immune system (**Figure 2B**). Additionally, we performed gene set enrichment analysis (GSEA) based on the KEGG and GO database of LN and CLE. All four analyses revealed a consistent upregulation in pathways linked to the upregulation of immune response, including systemic lupus erythematosus, B cell receptor signaling pathway, T cell activation, Th17 cell differentiation, and Type I interferon-mediated signaling pathway (**Figures 2C-F**). These observations reinforce the concept of a shared immunopathological pathway involving innate immunity. In addition, we also performed DO enrichment analysis. The results that both lupus nephritis and cutaneous lupus erythematosus were upregulated in DO enrichment analysis were used to validate these above results (**Figures 2G, H**).

### 3.3 Identification and validation of potential shared hub genes by machine learning models

**Figure 3:**
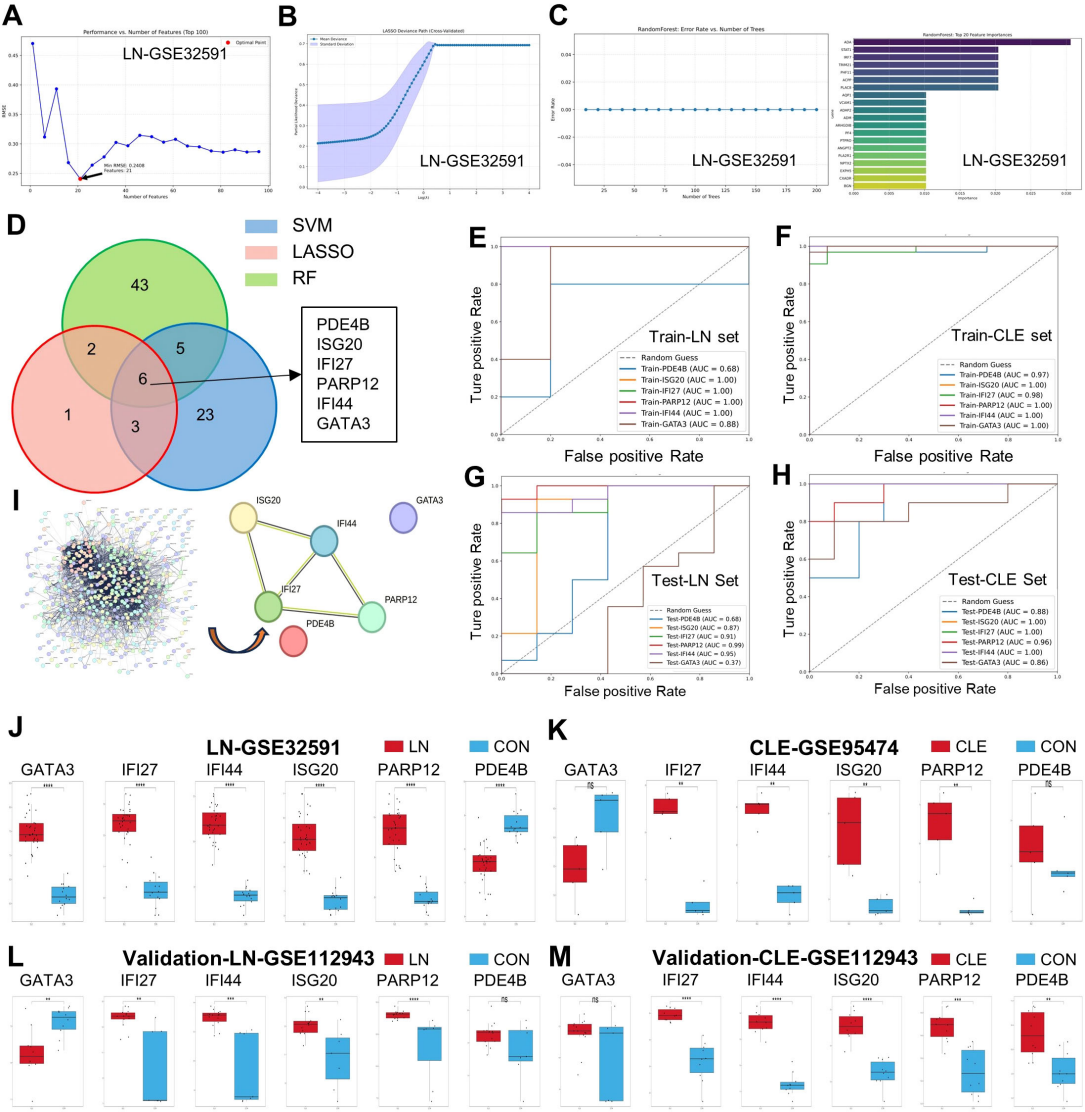
Construction and evaluation of machine learning models for hub gene selection. **(A)** Support vector machine (SVM) recursive feature elimination (RFE) curves showing the optimal number of features for LN (GSE32591). **(B)** Least absolute shrinkage and selection operator (LASSO) regression analysis in LN. **(C)** Random forest (RF) analysis of LN. **(D)** Venn diagram of overlapping hub genes identified by three machine learning algorithms (SVM, LASSO, and RF). **(E-H)** Receiver operating characteristic (ROC) curves evaluating the predictive performance of the selected genes in the training and testing sets of LN **(E, G)** and CLE **(F, H)**. **(I)** Protein–protein interaction (PPI) network of candidate genes constructed using the STRING database. Hub genes were identified by the MCC algorithm in Cytoscape, and the six genes (GATA3, IFI27, IFI44, ISG20, PARP12, PDE4B) are highlighted. **(J, K)** Box plots showing the expression of hub genes in LN (**J**, GSE32591) and CLE (**K**, GSE95474) training datasets compared with controls. **(L, M)** Validation of hub gene expression in an independent cohort (GSE112943) for LN **(L)** and CLE **(M)**. Data are presented as mean ± SEM. *p < 0.05, **p < 0.01, ***p < 0.001, ****p < 0.0001; ns, not significant.

To identify key genes that can predict concurrent renal involvement in SLE patients with cutaneous involvement, we selected the most important genetic features using multiple machine learning methods. Among the 497 candidate genes mentioned above, we sequentially performed further screening using Support Vector Machine (SVM), Least Absolute Shrinkage and Selection Operator (LASSO), and Random Forest (RF) (**Supplementary Table 4**). Using the SVM method, no irrelevant genes were filtered out from the candidate genes in the LN-GSE32591 and CLE-GSE95474 datasets, so all 497 genes were retained (**Figure 3A** and **Supplementary Figure 4A**). Through LASSO, 13 genes were selected from the candidate genes in the LN-GSE32591 and CLE-GSE95474 datasets (**Figure 3B** and **Supplementary Figure 4B**), while 92 genes were selected using RF from the candidate genes in the same datasets (**Figure 3C** and **Supplementary Figure 4C**). Furthermore, we further selected the gene features with a weight of ≥ 0.01 from the three algorithms to generate a Venn diagram, and finally identified 6 common putative biomarkers, namely PDE4B, ISG20, IFI27, PARP12, IFI44, and GATA3 (**Figure 3D**).

Furthermore, we evaluated the diagnostic predictive value of these core genes in the training set using ROC curves. In the Train-LN dataset, the AUC values were 0.68 for PDE4B, 1.00 for ISG20, 1.00 for IFI27, 1.00 for PARP12, 1.00 for IFI44, and 0.88 for GATA3 (**Figure 3E**). Similarly, in the Train-CLE dataset, the AUC values were 0.97 for PDE4B, 1.00 for ISG20, 0.98 for IFI27, 1.00 for PARP12, 1.00 for IFI44, and 1.00 for GATA3, all exceeding 0.7 (**Figure 3F**). These results indicate that these 6 genes exhibit good diagnostic performance and may serve as diagnostic putative biomarkers for predicting renal involvement in SLE patients with cutaneous involvement. In the validation sets, the AUC values from different cohorts also showed good predictive efficacy. In the Test-LN validation set, the AUC values for PDE4B, ISG20, IFI27, PARP12, IFI44, and GATA3 were 0.68, 0.87, 0.91, 0.99, 0.95, and 0.37, respectively. Except for PDE4B and GATA3 with AUC values less than 0.70, all other putative biomarkers had AUC values greater than 0.8 (**Figure 3G**). In the Test-CLE validation set, the AUC values for PDE4B, ISG20, IFI27, PARP12, IFI44, and GATA3 were 0.88, 1.00, 1.00, 0.96, 1.00, and 0.86, respectively, with all putative biomarkers showing AUC values greater than 0.8 (**Figure 3H**).

To further explore the interactions and expression patterns of the identified hub genes, we constructed a protein-protein interaction (PPI) network and analyzed gene expression across different datasets. The visualization of the PPI network highlights the roles of key predictive genes, and there are interactions among ISG20, IFI44, IFI27, and PARP12, indicating that they play core roles in molecular pathways related to LN and CLE (**Figure 3I**). Subsequently, box plots showed that in the training sets LN-GSE32591 and CLE-GSE95474, the four genes IFI27, IFI44, ISG20, and PARP12 were all significantly upregulated, while the variation trends of GATA3 and PDE4B were inconsistent (**Figures 3J, K**). More importantly, consistent differential trends were also observed in the validation sets LN-GSE112943 and CLE-GSE112943 (**Figures 3L, M**).

### 3.4 Single-cell analysis reveals cellular distribution of hub genes

**Figure 4:**
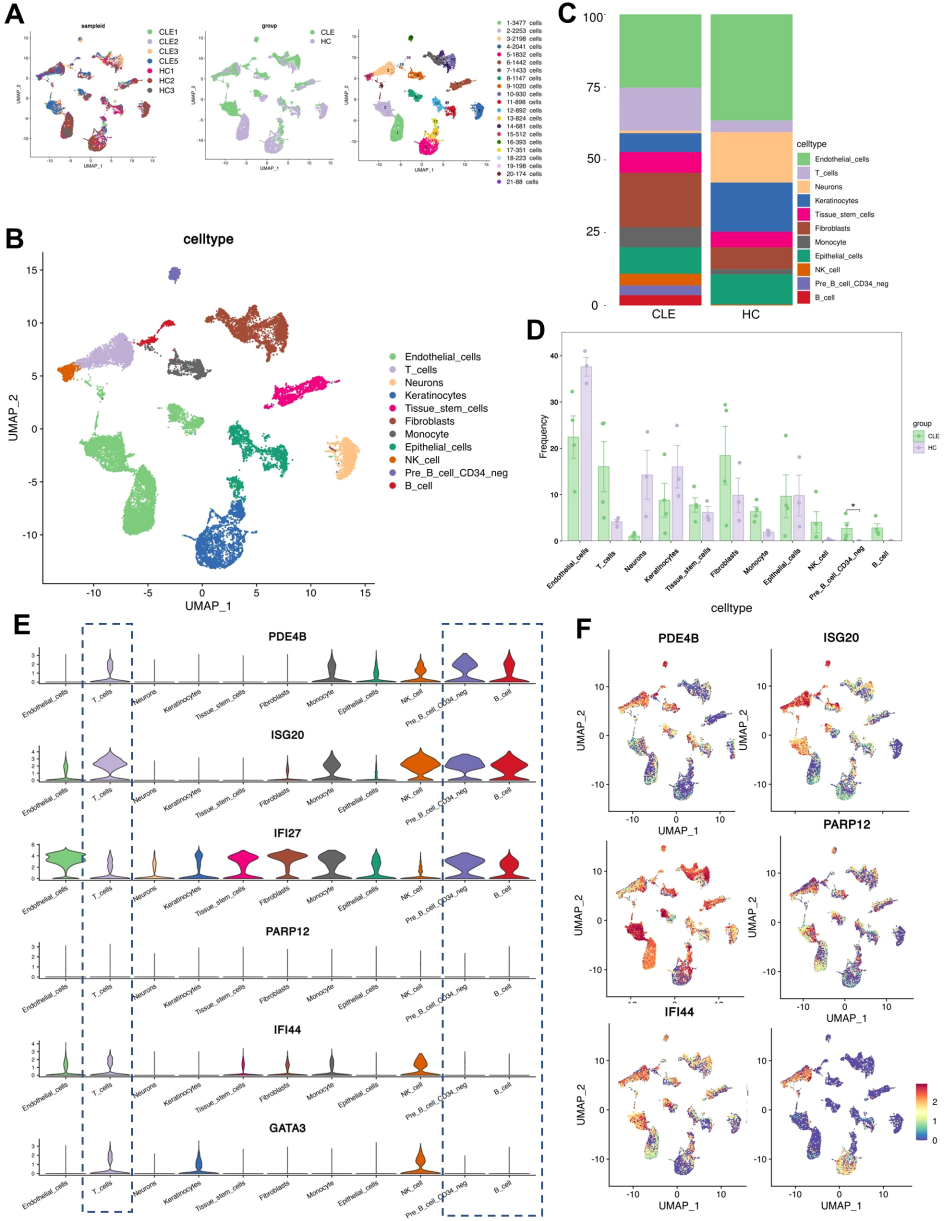
Validation of hub gene expression in the CLE single-cell dataset. **(A)** UMAP plots of cells from the CLE single-cell RNA-seq dataset,GSE294296,colored by sample group, and sample origin, cell type. **(B)** UMAP visualization showing the distribution of annotated immune and stromal cell populations. **(C)** Bar chart comparing cell-type composition between CLE patients and healthy controls (HC). **(D)** Quantification of relative frequencies of major immune cell subsets in CLE and HC groups. **(E)** Violin plots showing the expression of six hub genes (PDE4B, ISG20, IFI27, PARP12, IFI44, GATA3) across different cell types. **(F)** UMAP feature plots displaying the spatial expression of hub genes across the single-cell landscape.

To further investigate the cellular localization and expression characteristics of candidate hub genes, we analyzed a single-cell RNA sequencing dataset of CLE. As no suitable scRNA-seq dataset from LN kidney biopsies was available, the analysis was restricted to skin biopsy samples, including three healthy donors and five CLE patients, obtained from the GSE294296 dataset (**Table 1**, **Supplementary Figures 5A-I**). Uniform Manifold Approximation and Projection (UMAP) projections revealed distinct clustering of immune and non-immune cell subsets, including T cells, B cells, NK cells, monocytes, epithelial cells, endothelial cells, neurons, tissue stem cells, fibroblasts, and keratinocytes, with clear separation between CLE and healthy controls (HC) (**Figures 4A, B**). Comparison of cell composition showed altered proportions of multiple immune cell populations in CLE lesions relative to controls (**Figures 4C, D**), and we found the proportion of pre-B cells was significantly elevated in CLE relative to controls (**Figure 4D**). Moreover, both T-cell and B-cell compartments displayed an upward trend in the CLE group, although these changes did not reach statistical significance (**Figure 4D**).

Expression analysis at the single-cell level revealed that the hub genes PDE4B, ISG20, IFI27, PARP12, IFI44, and GATA3 were differentially distributed across immune and non-immune cell populations. Violin plots demonstrated that PDE4B, ISG20, and IFI27 were highly expressed in most cell subsets, particularly in T cells, NK cells, and B cells (**Figure 4E**). Functionally, PDE4B regulates cAMP-dependent inflammatory signaling, with aberrant activity contributing to immune dysregulation. ISG20 acts as a type I interferon–inducible exonuclease essential for innate antiviral defense. And IFI27 serves as a sensitive marker of type I interferon pathway activation in autoimmune diseases. Their aberrant expression underscores the interplay between dysregulated cAMP signaling and persistent interferon-driven immune activation, processes central to the pathogenesis of autoimmune disorders such as SLE and CLE. UMAP feature plots further validated the cell-type–specific expression profiles, showing strong enrichment of interferon-stimulated genes (e.g., IFI27, IFI44, ISG20, PARP12) within immune cell subsets, whereas GATA3 and PDE4B displayed broader but distinct expression patterns (**Figure 4F**).

Additionally, we analyzed SLE peripheral immune microenvironment by using SLE PBMC dataset (GSE135779) (**Table 1**). The analysis revealed significant effects (**Supplementary Figure 6A**). UMAP technique delineated eight distinct cell clusters, each represented by unique color codes (**Supplementary Figure 6A**). Cell-type classification was conducted utilizing the SingleR package, which categorized the clusters into nine primary immune cell types based on the expression of established marker genes: CD4^+^ T cells, CD4^+^ effector memory T cells (CD4^+^Tem), CD8^+^ T cells, CD8^+^ effector memory T cells (CD8^+^Tem), monocytes, natural killer (NK) cells, naïve B cells, and megakaryocyte-erythroid progenitors (MEPs) (**Supplementary Figure 6B**). A comparative assessment of the proportions of these cell types demonstrated a significant decrease in the population of naïve B cells, alongside a downward trend in CD4^+^ T cells within SLE samples. Conversely, monocytes and CD8^+^ T cells exhibited a slight increase, although this was not statistically significant (**Supplementary Figures 6C, D**).

### 3.5 Validation of immune dysregulation in the LPR mouse model

**Figure 5:**
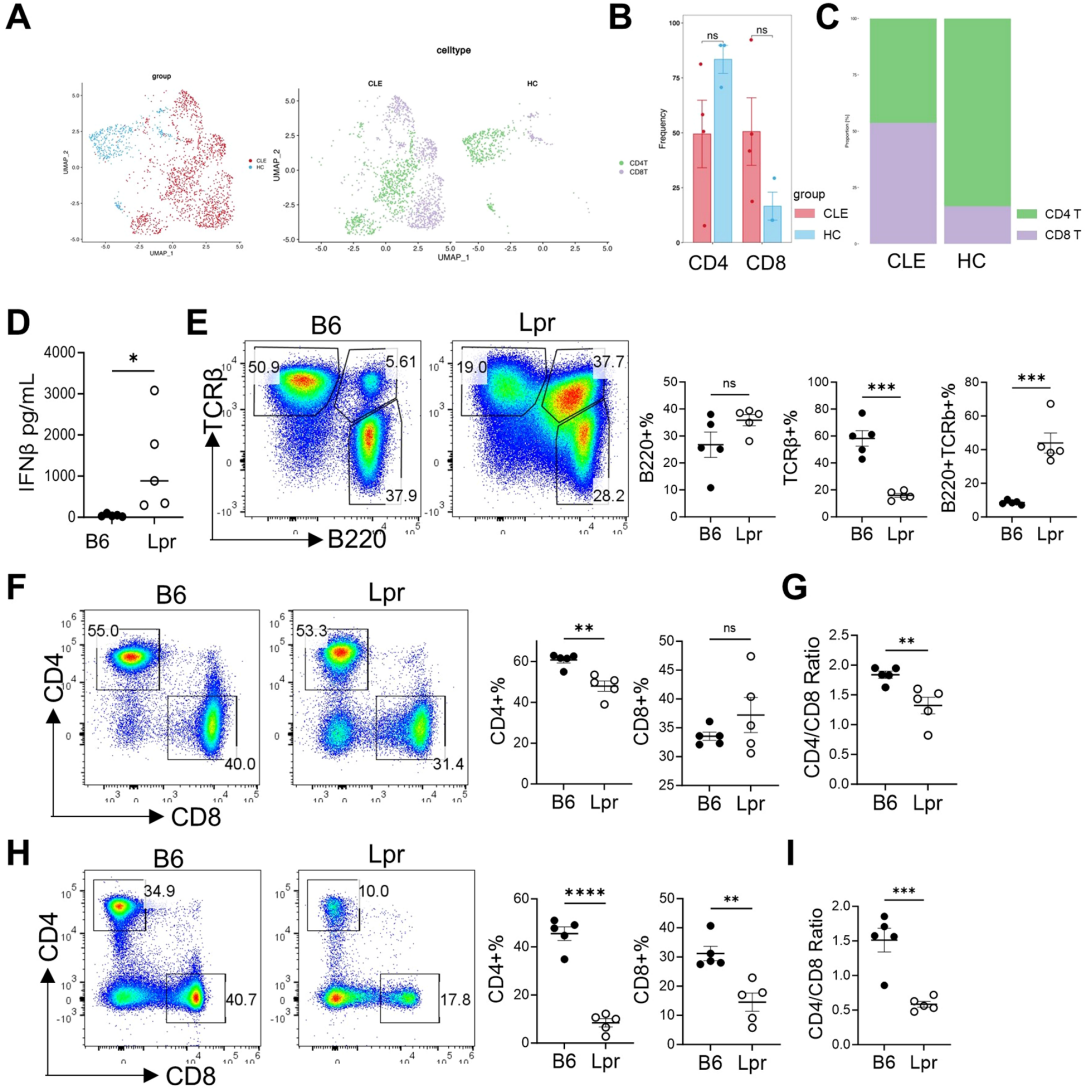
*In vivo* validation by using LPR mouse model. **(A)** UMAP visualization of immune cell subsets (CD4^+^ and CD8^+^ T cells) in CLE and HC groups from GSE294296 dataset. **(B)** Frequency of CD4^+^ and CD8^+^ T cells in CLE and HC groups. **(C)** Distribution of CD4^+^ and CD8^+^ T cells between CLE and HC group0s. **(D)** Serum IFN-β levels in B6 and LPR mice. **(E)** Flow cytometry analysis of B220^+^ B cells, TCRβ^+^ T cells and B220^+^TCRβ^+^ cells from lymph nodes with corresponding percentage. **(F)** Representative flow cytometry plots and statistical analysis of CD4^+^ and CD8^+^ T cells from TCRβ^+^ T cells from lymph nodes. **(G)** CD4/CD8 T-cell ratio in B6 and LPR mice from lymph nodes. **(H)** Flow cytometry analysis of peripheral blood CD4^+^ and CD8^+^ T cells and corresponding percentage in B6 and LPR mice. **(I)** CD4/CD8 ratio in peripheral blood in B6 and LPR mice. Data are presented as mean ± SEM. *p < 0.05, **p < 0.01, ***p < 0.001; ns, not significant.

To further investigate immune dysregulation in lupus, with a particular focus on CD4^+^ and CD8^+^ T-cell populations, we first analyzed CLE immune cells using UMAP. This analysis revealed distinct clustering patterns of CD4^+^ and CD8^+^ T cells between CLE patients and healthy controls (**Figure 5A****).** Quantitative assessment showed a decreasing trend in CD4^+^ T-cell proportions and a concomitant increasing trend in CD8^+^ T cells in CLE, although there were no statistical significance, resulting in an overall reduction of the CD4/CD8 ratio in the CLE patients group (**Figures 5B, C****).**

To further validate these findings *in vivo*, we employed the Lpr lupus mouse model and B6 control mice. Consistent with the bioinformatic analyses above, serum IFN-β levels were significantly elevated in Lpr mice (**Figure 5D**). Flow cytometry analysis further confirmed profound immune perturbations in LPR mice. The proportion of total TCRβ^+^ T cells was significantly reduced in Lpr, whereas the TCRβ^+^B220^+^ population, representing the aberrant T cells characteristic of C57BL/6-FasLpr mice, was markedly increased (12, 13). In contrast, the proportion of B220^+^ B cells remained unchanged (**Figure 5E**).

Within splenic T cell populations, CD4^+^ T cells were significantly decreased, while CD8^+^ T cells showed no significant difference, leading to a pronounced reduction in the CD4/CD8 ratio in Lpr mice. This alteration mirrored the pattern observed in the CLE patient group (**Figures 5C, F, G**). Similarly, in peripheral blood, both CD4^+^ and CD8^+^ T-cell frequencies were decreased in Lpr mice; however, the reduction in CD4^+^ T cells was more pronounced, again resulting in a significantly decreased CD4/CD8 ratio in Lpr (**Figures 5H, I****).** Collectively, these results indicate that the Lpr mouse model recapitulates key features of immune dysregulation observed in lupus, characterized by enhanced type I interferon signaling and disrupted CD4^+^/CD8^+^ T-cell homeostasis.

### 3.6 Analysis of immune cell infiltration and correlation of biomarkers with infiltrating immune cells

**Figure 6:**
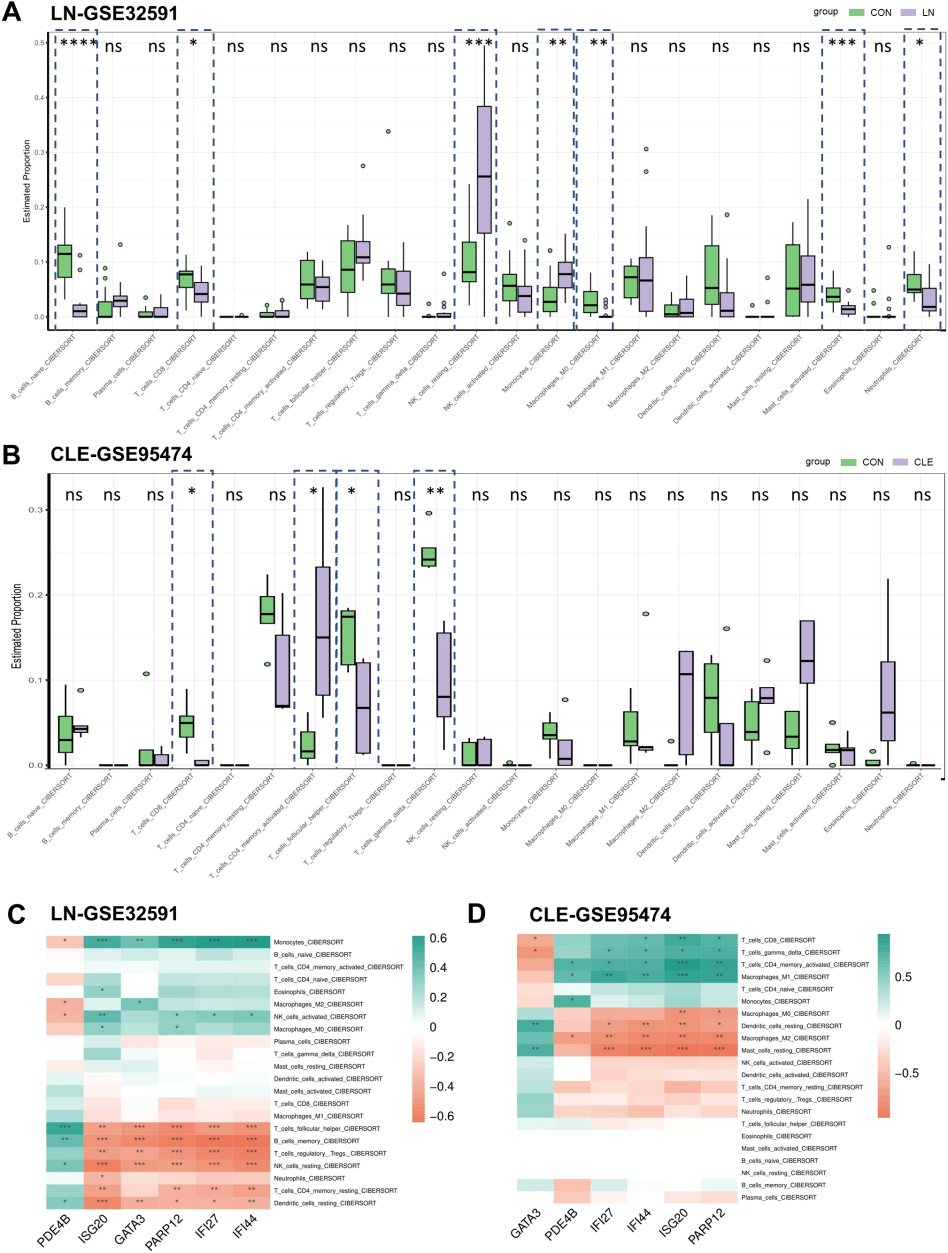
Immune cell infiltration analysis in LN and CLE. **(A, B)** Comparison of 22 immune cell subtypes in the LN-GSE32591 cohort and the CLE-GSE95474 cohort. Red color represents LN/CLE patients and blue color represents normal people. **(C, D)** Heatmaps showing the correlation between hub genes and immune cells. Green color represents positive correlation and red color represents negative correlation. Data are presented as mean ± SEM. *p < 0.05, **p < 0.01, ***p < 0.001, ****p < 0.0001; ns, not significant.

To explore shared and tissue-specific immune features between LN and CLE, immune cell infiltration was estimated using the CIBERSORT algorithm. LN samples exhibited significantly altered proportions of multiple immune cell subsets compared with controls, including increased fractions of memory B cells, follicular helper T cells, regulatory T cells, activated NK cells, and monocytes, indicating a broadly activated immune microenvironment within the kidney (**Figure 6A**). In contrast, CLE samples displayed a distinct immune infiltration pattern. CLE lesions were characterized by increased proportions of γδ T cells, macrophage subsets (M1 and M2), and resting mast cells, suggesting enhanced innate immune activation and inflammatory remodeling in cutaneous tissue (**Figure 6B**). Together, these findings highlight pronounced organ-specific immune heterogeneity in lupus pathogenesis.

To explore the immunological relevance of the identified hub genes, correlation analyses were performed between hub gene expression and infiltrating immune cell fractions. In LN, expression levels of PDE4B, ISG20, IFI27, PARP12, IFI44, and GATA3 were significantly associated with multiple immune cell populations, particularly monocytes, macrophages, T-cell subsets, and NK cells (**Figure 6C**). Notably, interferon-related genes (ISG20, IFI27, IFI44, and PARP12) showed strong positive correlations with pro-inflammatory myeloid cells, supporting their involvement in interferon-driven immune activation within the kidney. Distinct correlation patterns were observed in CLE samples, where hub gene expression was more closely linked to macrophage subsets, dendritic cells, and CD4^+^, CD8^+^, and γδ T cells (**Figure 6D****).** These results indicate that although LN and CLE share core pathogenic genes, their functional associations with immune cell infiltration differ across tissues, reflecting organ-specific immune remodeling in lupus.

## 4 Discussion

Lupus nephritis (LN) and cutaneous lupus erythematosus (CLE) are two significant autoimmune disorders, each with distinct clinical features yet arising from overlapping immunopathogenic mechanisms. They present considerable clinical challenges and affect a substantial proportion of individuals diagnosed with systemic lupus erythematosus (SLE) (14). LN causes kidney inflammation that can lead to renal dysfunction (15, 16), while CLE presents with diverse skin lesions that severely affect patients’ quality of life (17). CLE primarily manifests as cutaneous inflammation driven by aberrant immune responses and heightened type I interferon signaling (18). Importantly, about 50% of individuals with SLE develop some form of renal involvement. This highlights the urgent need for prompt diagnosis and effective management to prevent severe complications and reduce the economic burden of chronic care (3). Although current therapies such as corticosteroids and immunosuppressants are available, their limitations create a pressing need to discover novel biomarkers and therapeutic targets to improve patient outcomes (11, 19).

The objective of this investigation is to elucidate the expression profiles of particular genes associated with both LN and CLE. We employ advanced bioinformatics methodologies and machine learning techniques to identify putative biomarkers. Our research extends previous findings that indicates a common immune dysregulation in both LN and CLE, thereby suggesting that a comprehensive understanding of these molecular pathways is critical for effective diagnosis and treatment. Through careful analysis of bulk transcriptome data, single-cell RNA sequencing, and machine learning, we identified overlapping differentially expressed genes and putative biomarkers predicting renal involvement in SLE patients with cutaneous manifestations. These findings were further validated in a lupus-prone mouse model. Our results reveal a convergent axis of type I interferon signaling activation and cAMP-dependent inflammatory pathways, accompanied by impaired T-cell regulation, that underlies the comorbid pathogenesis of LN and CLE. This shared molecular signature highlights specific gene modules that may serve as both putative shared molecular signatures and hypothesis-generating therapeutic targets. The ensuing discussion will examine the implications of our main findings and their potential impact on future research and clinical practice (20, 21).

This investigation performed a differential gene expression analysis, uncovering a substantial number of differentially expressed genes (DEGs) in both lupus nephritis (LN) and cutaneous lupus erythematosus (CLE) patient groups. These findings reflect the intricate molecular framework of these disorders. In particular, the analysis revealed 361 DEGs in the LN group and 711 DEGs in the CLE group, with 99 genes which were common to both cohorts. This observation underscores significant alterations in gene expression that may contribute to the pathogenesis of these conditions. The results not only provide critical insights into the immunopathological mechanisms that characterize LN and CLE, but also suggest that certain DEGs may serve as putative molecular indicators associated with disease activity or treatment response, warranting further validation in independent cohorts. The unique patterns of gene upregulation and downregulation detected in these cohorts suggest a complex interplay between immune system dysregulation and the clinical manifestations of the diseases, indicating a need for further exploration of these DEGs in relation to clinical characteristics (21). Understanding the specific roles of these gene expression alterations could pave the way for identifying novel therapeutic targets, ultimately improving treatment strategies for patients with these complex autoimmune diseases (22, 23).

This study uses weighted gene co-expression network analysis (WGCNA) to clarify the relationship between gene expression profiles and clinical features in lupus nephritis (LN) and cutaneous lupus erythematosus (CLE). By identifying ten distinct modules in the LN dataset and 23 in the CLE dataset, especially those linked to clinical traits, we highlight the complex interplay between gene expression and disease manifestations. Importantly, modules associated with immune response pathways reveal a common disruption of immune functions in both diseases, enabling targeted studies on their roles. Future research should examine the biological relevance of these modules, their roles in disease progression, and the potential for targeting them therapeutically (24). Furthermore, understanding how interactions within these modules change over time may shed light on disease progression and treatment responses. This knowledge could ultimately aid in developing better management strategies for patients with LN and CLE.

The analysis of functional enrichment provides deeper insights into the roles of immune-related pathways in lupus nephritis (LN) and cutaneous lupus erythematosus (CLE). It highlights the presence of common immunopathological features in both conditions. The discovery of enriched pathways, such as interferon-gamma and type I interferon signaling, underscores the activation of innate immune responses in these disorders. This finding suggests that targeting these pathways could offer new treatment options, especially since current therapies often fail in some patients. These pathways are relevant to established lupus therapies. This highlights the urgent need for further studies to confirm their therapeutic effectiveness in clinical settings (25). Future research should focus on identifying immune pathways as targets for intervention, to help develop personalized treatments for patients with LN and CLE (26).

Recent transcriptomic and single-cell studies have revealed that LN and CLE share strikingly similar molecular features. Arazi et al. demonstrated that both kidney and skin lesions exhibit strong type I interferon activation and immune cell infiltration, underscoring interferon signaling as a central pathogenic axis (27). Der et al. further confirmed the presence of conserved interferon-driven immune signatures across tissues and identified overlapping gene modules with potential diagnostic and therapeutic relevance (14). Together, these findings provide compelling evidence that LN and CLE share common immunopathogenic mechanisms, highlighting putative shared molecular signatures and hypothesis-generating therapeutic targets that transcend organ-specific manifestations.

Machine learning has become a powerful tool to identify putative biomarkers in autoimmune diseases. This study identified six key biomarkers with promising diagnostic performance, offering new opportunities to enhance patient management. The area under the curve (AUC) values that surpass 0.7 in both training and validation datasets indicate these biomarkers, including PDE4B, ISG20, IFI27, PARP12, IFI44 and GATA3, have potential for clinical use in predicting renal involvement in SLE patients with cutaneous symptoms. Further research is critical to explore the clinical applicability of these biomarkers. Future studies should assess their long-term predictive power and feasibility for inclusion in standard diagnostic protocols (3, 25). Integrating these biomarkers could lead to more personalized therapies, improving patient outcomes and quality of life for those with lupus.

Among the six hub genes, PDE4B is particularly notable because it encodes a cAMP-specific phosphodiesterase that degrades intracellular cAMP and thereby restrains PKA/CREB anti-inflammatory signaling. Excessive PDE4B activity lowers cAMP levels, unleashing NF-κB–mediated cytokine production and amplifying inflammatory responses (28, 29). Relevantly for lupus, pharmacologic PDE4 inhibition (NCS 613) increases cAMP, suppresses systemic inflammation, and reduces immune-complex deposition in the kidneys of MRL/lpr lupus-prone mice, supporting a cAMP-dependent inflammatory axis in LN pathogenesis (30).

Within this inflammatory axis, interferon-inducible chemokines emerge as key drivers, consistent with chronic type I interferon signaling being a hallmark of SLE pathogenesis. In our study, ISG20 was identified as one of the most robustly upregulated genes across both datasets, functioning as a critical effector of interferon-mediated antiviral defense (31). Beyond its canonical antiviral role, ISG20 is strongly induced in glomerular endothelial cells through TLR3 signaling, where it promotes chemokine production and glomerular inflammation, thereby contributing to lupus nephritis pathogenesis (32). Its consistent upregulation in multiple immune cell subsets of SLE highlights a central role in sustaining interferon-driven immune dysregulation and reinforces ISG20 as a potential cross-tissue biomarker and therapeutic target (32).

Among interferon-stimulated genes, IFI27 acts as a mitochondrial protein that mediates IFN-α-induced apoptosis via rapid cytochrome C release and activation of caspases (33). It also interacts with cytosolic RNA sensors, such as MDA5, inhibiting their oligomerization and dampening innate immune activation (32, 34). In SLE settings, IFI27 has been proposed as a diagnostic biomarker correlated with disease activity (35). Together, these roles position IFI27 as a pivotal node linking interferon activation, programmed cell death, and immune regulation in lupus pathogenesis.

In parallel, PARP12 represents an interferon-inducible member of the poly (ADP-ribose) polymerase family that regulates RNA stability and translation, amplifying interferon-stimulated gene networks and sustaining the chronic interferon signature observed in lupus (36). IFI44 is a canonical ISG whose elevated expression is strongly correlated with disease activity and flares in SLE, making it a well-established marker of interferon signature intensity (37). Finally, GATA3, classically a master regulator of Th2 differentiation, is also implicated in lupus: studies have observed altered GATA3 mRNA expression and Th1/Th2 transcription factor imbalance in SLE peripheral blood (38).

Accumulating evidence suggests that lupus-associated tissue damage arises from interconnected inflammatory and type I interferon–driven pathways rather than isolated organ-specific mechanisms (14). In this context, the hub genes identified in our study—PDE4B, ISG20, and IFI27—appear to converge on a shared pathogenic axis linking cutaneous inflammation to renal involvement. PDE4B encodes a cAMP-specific phosphodiesterase that degrades intracellular cAMP, thereby attenuating PKA/CREB-mediated anti-inflammatory signaling and potentiating NF-κB–dependent cytokine production (28). Aberrant upregulation of PDE4B in immune cells has been shown to lower the inflammatory threshold and promote systemic immune activation, while pharmacological inhibition of PDE4 ameliorates immune complex deposition and renal inflammation in lupus-prone mice (30). In parallel, ISG20 and IFI27 represent core components of the type I interferon program, a central hallmark of SLE. ISG20 functions as an interferon-inducible exonuclease that amplifies interferon-driven immune responses and chemokine production, thereby sustaining immune cell recruitment and inflammatory signaling (31). Notably, ISG20 is strongly induced in glomerular endothelial cells and contributes to chemokine-mediated leukocyte infiltration in lupus nephritis, implicating it directly in renal pathology (32). IFI27, a mitochondria-associated interferon-inducible protein, has been implicated in IFN-α–induced mitochondrial apoptosis and cellular stress responses, linking chronic interferon signaling to immune cell dysfunction and tissue injury (33). Its elevated expression has also been proposed as a biomarker of disease activity in lupus (35). Together, these findings support a mechanistic model in which dysregulated cAMP signaling cooperates with persistent interferon activation to drive T-cell and myeloid cell dysfunction, facilitating immune cell trafficking, tissue infiltration, and sustained inflammatory crosstalk between skin and kidney (**Supplementary Figure 7**). This integrated framework provides a biological basis for the shared molecular signatures observed in LN and CLE and highlights therapeutic opportunities targeting upstream regulatory nodes rather than individual organ manifestations.

Both LN and CLE are driven by shared yet tissue-context–dependent immune mechanisms. Type I interferon signaling represents a central pathogenic axis in both organs, as demonstrated by transcriptomic and single-cell studies showing robust interferon-stimulated gene activation in renal and cutaneous lesions (14). However, downstream immune effector pathways diverge across tissues. LN is characterized by prominent myeloid cell activation and immune complex–mediated inflammation within the kidney, accompanied by dysregulated T-cell responses and aberrant B-cell activation (15). In contrast, CLE lesions display enhanced innate immune activation, including macrophage infiltration and γδ T-cell expansion, alongside sustained interferon-driven epidermal inflammation (18). Beyond interferon signaling, our findings highlight immunometabolic dysregulation as a potential contributor to organ-specific pathology. In particular, PDE4B-mediated cAMP signaling has been implicated in amplifying NF-κB–dependent inflammatory responses, and pharmacologic PDE4 inhibition has been shown to attenuate renal immune injury in lupus-prone mice (30). These observations suggest that metabolic–inflammatory pathways may act in concert with interferon signaling to shape tissue-specific immune responses in LN and CLE.

The knowledge acquired from the analysis of single-cell RNA sequencing provides a deeper comprehension of the cellular environment in lupus. It emphasizes unique subsets of immune and non-immune cells that most of the shared hub genes are highly expressed in T cell, B cell, and NK cell. Variations in biomarker expression across different immune cell populations highlight the heterogeneity of the immune response in lupus nephritis (LN) and cutaneous lupus erythematosus (CLE). These variations may influence disease outcomes and treatment efficacy. Prior studies have shown expansion and altered function of these cells in both diseases, supporting the idea that shared immune phenotypes underlie diverse clinical manifestations (14). These observations suggest that specific immune cell populations play a pivotal role in the pathogenesis of these disorders, indicating the need for further investigation into their functions. In addition, the prospective application of single-cell analysis may extend beyond lupus to address other autoimmune conditions, potentially uncovering shared mechanisms and therapeutic targets across various diseases. Understanding the impact of immune cell diversity on disease progression and treatment responses is essential for developing more effective therapies for autoimmune disorders. This underscores the importance of advanced methodologies in elucidating the complexities of immune dysregulation.

Our *in vivo* validation using the C57BL/6-FasLpr lupus mouse model provided supportive physiological evidence for the T-cell perturbations suggested by the human transcriptomic data. Lpr mice exhibited significantly elevated serum IFN-β levels relative to B6 controls, supporting the role of systemic interferon activation in disease. Flow cytometric analyses showed a T-cell–centric immune disturbance. Within the lymph nodes, a higher CD4/CD8 ratio was found in Lpr mice; a similar pattern was observed in peripheral blood. These findings highlight an imbalance in T-cell homeostasis in SLE, consistent with prior reports of T-cell dysregulation in lupus (39, 40). These findings are aligned with previous reports describing disrupted T-cell homeostasis in lupus and reinforce immune-phenotype directionality across species. Importantly, we now interpret the murine data as supportive contextual evidence rather than mechanistic gene-level validation, and we acknowledge that causal inference for the identified hub genes will require targeted perturbation studies (e.g., knockdown or over-expression) in future work. This reframed interpretation avoids overstatement and positions the animal findings as a physiological complement to the computational analyses.

Consistent with the transcriptomic signatures, CIBERSORT analysis revealed that both LN and CLE are characterized by an activated yet tissue-specific immune landscape. In LN kidneys, we observed increased proportions of memory B cells, follicular helper T cells, regulatory T cells, activated NK cells and circulating monocytes, indicating a milieu that favors autoreactive B-cell help, effector T-cell expansion and myeloid-driven inflammation. In contrast, CLE lesions were dominated by γδ T cells, M1/M2 macrophages and resting mast cells, reflecting the prominence of innate and barrier-associated immunity in cutaneous tissue. Rather than representing conflicting results, these patterns likely reflect the distinct microenvironments of glomerular versus epidermal/dermal compartments, while still converging on common pathways of interferon-driven myeloid and T-cell activation. Supporting this notion, interferon-stimulated hub genes (ISG20, IFI27, IFI44, PARP12) showed positive correlations with monocytes/macrophages and activated T-cell subsets in both datasets, suggesting that these genes participate in a shared axis linking gene-level dysregulation to immune-cell recruitment and tissue injury. Together, the integrated gene–cell–tissue view provided by the immune-infiltration and correlation analyses supports a model in which organ-specific immune compositions are superimposed on a conserved systemic program of interferon-enhanced myeloid and T-cell activation in lupus.

## 5 Limitations

Several methodological and interpretive constraints should be acknowledged. The reliance on small, publicly deposited datasets—without independent confirmation in external cohorts—raises the risk of overfitting and limits the generalizability of the proposed molecular signatures. The CLE cohort is particularly underpowered. In addition, the lack of matched scRNA-seq profiling from lupus nephritis kidneys prevents resolution of tissue-specific immune microenvironments and precludes mechanistic inference in renal compartments. Given the retrospective design and heterogeneous clinical annotation, the machine-learning outputs should be interpreted strictly as statistical prioritization of putative candidate genes rather than diagnostic or predictive biomarkers. Any clinical translation will require validation in larger, prospectively enrolled cohorts, ideally incorporating functional experiments and therapeutic interrogation.

## 6 Conclusion

This study identifies cross-tissue molecular features shared by LN and CLE, and delineates a working model in which cAMP-dependent inflammatory signaling and interferon-stimulated gene activation may contribute to disease in both skin and kidney. By integrating bulk transcriptomic analysis, statistical gene prioritization, and single-cell profiling, the findings highlight immune pathways and candidate genes that merit further mechanistic exploration. These observations are intended as hypothesis-generating leads rather than validated diagnostic markers, and their relevance for patient stratification or therapeutic targeting will require confirmation in prospectively designed clinical cohorts and experimental studies.

## Data Availability

The RNA-seq databases were analyzed from GEO dataset, including GSE32591 (https://www.ncbi.nlm.nih.gov/geo/query/acc.cgi?acc=GSE32591), GSE95474 (https://www.ncbi.nlm.nih.gov/geo/query/acc.cgi?acc=GSE95474), GSE112943 (https://www.ncbi.nlm.nih.gov/geo/query/acc.cgi?acc=GSE112943). The analysis of protein–protein interactions (PPI) was analyzed from STRING database (https://string-db.org/). The scRNA-seq database was analyzed from GEO dataset GSE294296 (https://www.ncbi.nlm.nih.gov/geo/query/acc.cgi?acc=GSE294296) and GSE135779 (https://www.ncbi.nlm.nih.gov/geo/query/acc.cgi?acc=GSE135779). The datasets generated for this study can be found in the article.

## Supplementary material

Supplementary Figure 1 Flow diagram of this research.

Supplementary Figure 2 Gene significance (GS) and module membership (MM) within the modules. **(A-F)** Scatter plots with |r| > 0.3 in GSE32591. **(G-M)** Scatter plots with |r| > 0.3 in GSE95474.

Supplementary Figure 3 Functional enrichment analyses of DEGs in LN and CLE datasets. **(A, B)** KEGG pathway enrichment analysis of differentially expressed genes (DEGs) in LN (**A**, GSE32591) and CLE (**B**, GSE95474). The top 20 enriched pathways are shown. Dot size indicates the number of enriched genes, and color reflects adjusted p-values. **(C, D)** GO enrichment analysis of DEGs in LN (**C**, GSE32591) and CLE (**D**, GSE95474). The top 30 enriched GO terms are presented, grouped into biological process (green), cellular component (orange), and molecular function (blue).

Supplementary Figure 4 Construction and evaluation of machine learning models for hub gene selection for CLE. **(A)** Support vector machine (SVM) recursive feature elimination (RFE) curves showing the optimal number of features for CLE (GSE95474). **(B)** Least absolute shrinkage and selection operator (LASSO) regression analysis in CLE. **(C)** Random forest (RF) analysis of CLE.

Supplementary Figure 5 Quality control and identification of skin cell populations in Single-Cell Data of CLE. **(A, B)** Quality control of peripheral blood mononuclear cells (PBMCs) from CLE patients and healthy controls, showing the dataset before **(A)** and after **(B)** the exclusion of low-quality cells during the initial filtering process. **(C)** Heatmap illustrating the expression of canonical marker genes across identified cell populations. **(D-I)** Differential analysis of hub genes based on single-cell data. Data are presented as mean ± SEM. *p < 0.05, **p < 0.01, ***p < 0.001, ****p < 0.0001; ns, not significant.

Supplementary Figure 6 PBMC Single-cell dataset validates key gene expression. **(A)** Data integration, normalization, and standardization across all samples, and Uniform Manifold Approximation and Projection (UMAP) clustering into 8 clusters. **(B)** UMAP plot of all single cells, annotated by major cell types with distinct color coding in SLE single-cell dataset. **(C)** Bar chart showing the distribution of major immune cell subsets between SLE patients and healthy donor (HD). **(D)** Frequency of each cell subsets in SLE and HD.

Supplementary Figure 7 Integrated mechanistic model linking cutaneous inflammation and renal involvement in lupus. In cutaneous lupus lesions, type I interferon activation triggers upregulation of interferon-stimulated genes such as ISG20 and IFI27, promoting chemokine production, immune cell recruitment, and mitochondrial stress responses. In parallel, increased PDE4B expression reduces intracellular cAMP, attenuates PKA/CREB activity, and enhances NF-κB–dependent inflammatory signaling, thereby lowering the threshold for immune activation. These molecular perturbations converge to drive dysregulated T-cell and myeloid-cell responses, facilitating trafficking of effector lymphocytes and inflammatory monocytes/macrophages from the skin into the circulation and ultimately into the kidney. The resulting immune infiltration contributes to glomerular inflammation and tissue injury, offering a mechanistic basis for the shared transcriptomic signatures observed in CLE and LN.

Supplementary Table 1 GSE32591 dataset.

Supplementary Table 2 GSE95474 dataset.

Supplementary Table 3 The shared genes of DEG and WGCNA.

Supplementary Table 4 Machine-learning feature weights by model.

Supplementary Table 5 Machine-learning model parameters and optimization settings.
