# Supplementary material for: Cross-tissue integrative transcriptomic and multimodal analyses suggest shared immune signatures linking lupus nephritis and cutaneous lupus erythematosus
Source: Front Immunol. 2026 Feb 24;17:1716516. doi: 10.3389/fimmu.2026.1716516 (PMC12971934; doi:10.3389/fimmu.2026.1716516)
Supplement: Supplementary Figure 1 — Flow diagram of this research. [file Image1.pdf]

Supplementary Material

1 Supplementary Figures

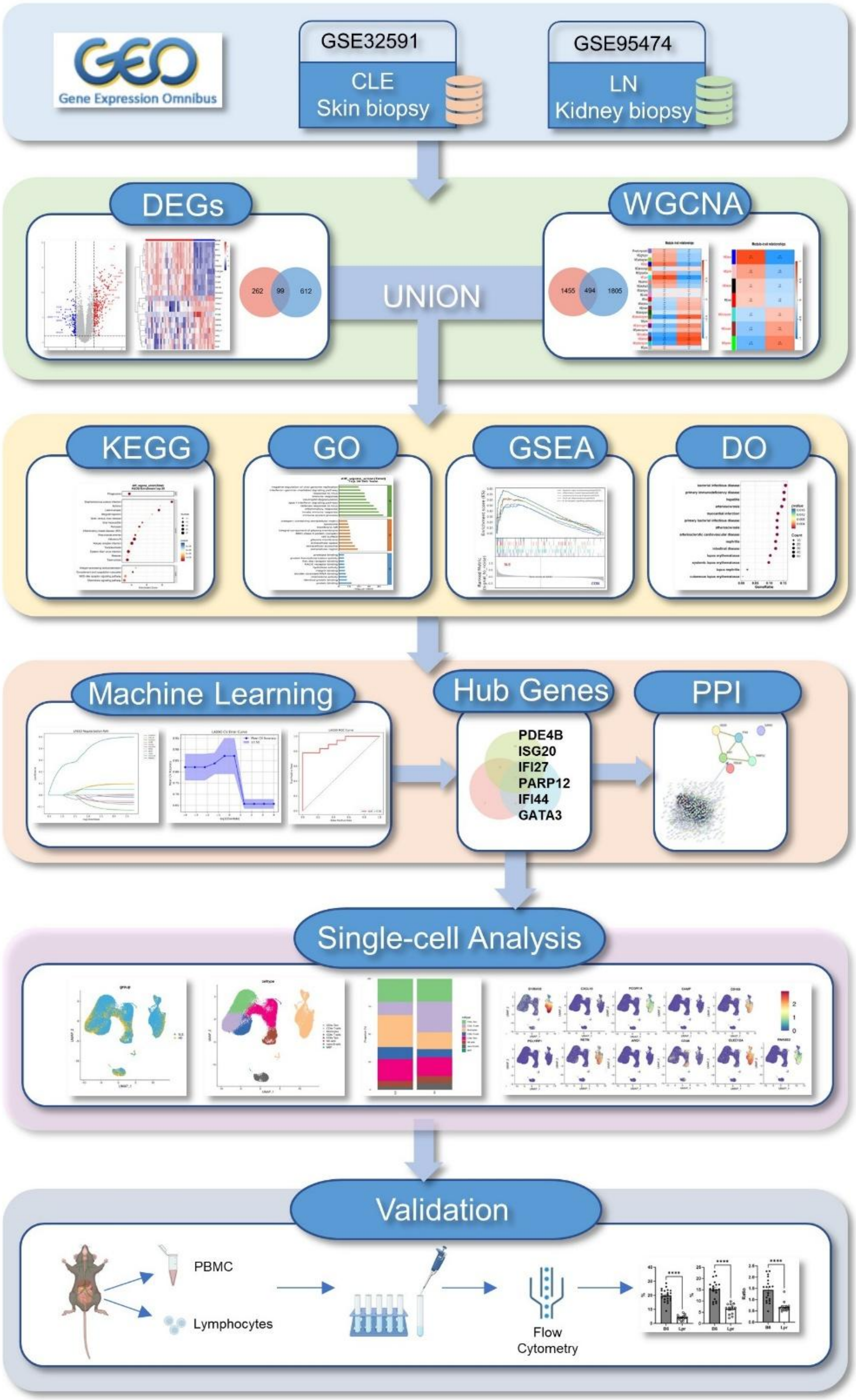

Supplementary Figure 1. Flow diagram of this research.

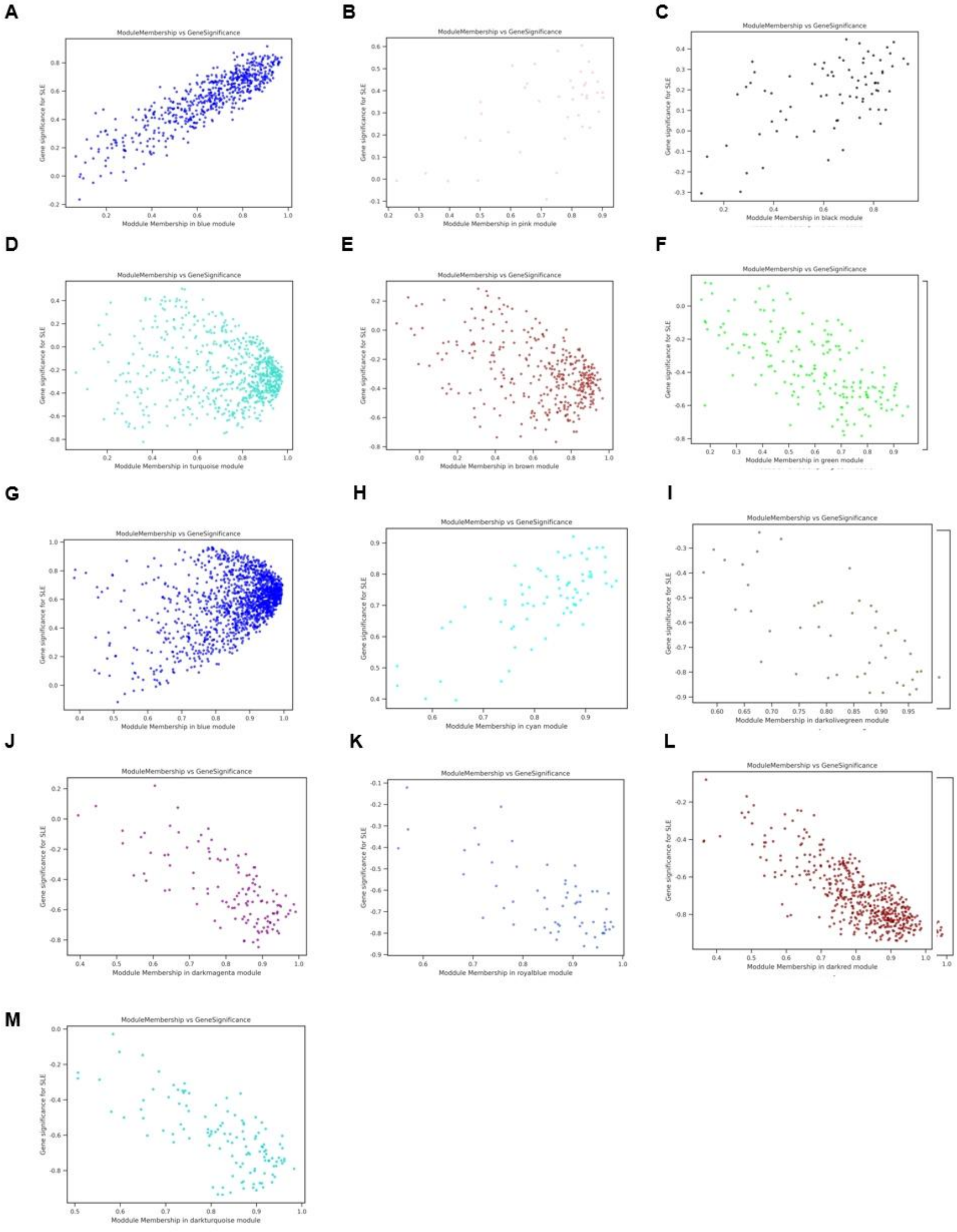

Supplementary Figure 2. Gene significance (GS) and module membership (MM) within the modules. (A-F) Scatter plots with |r| > 0.3 in GSE32591. (G-M) Scatter plots with |r| > 0.3 in GSE95474.

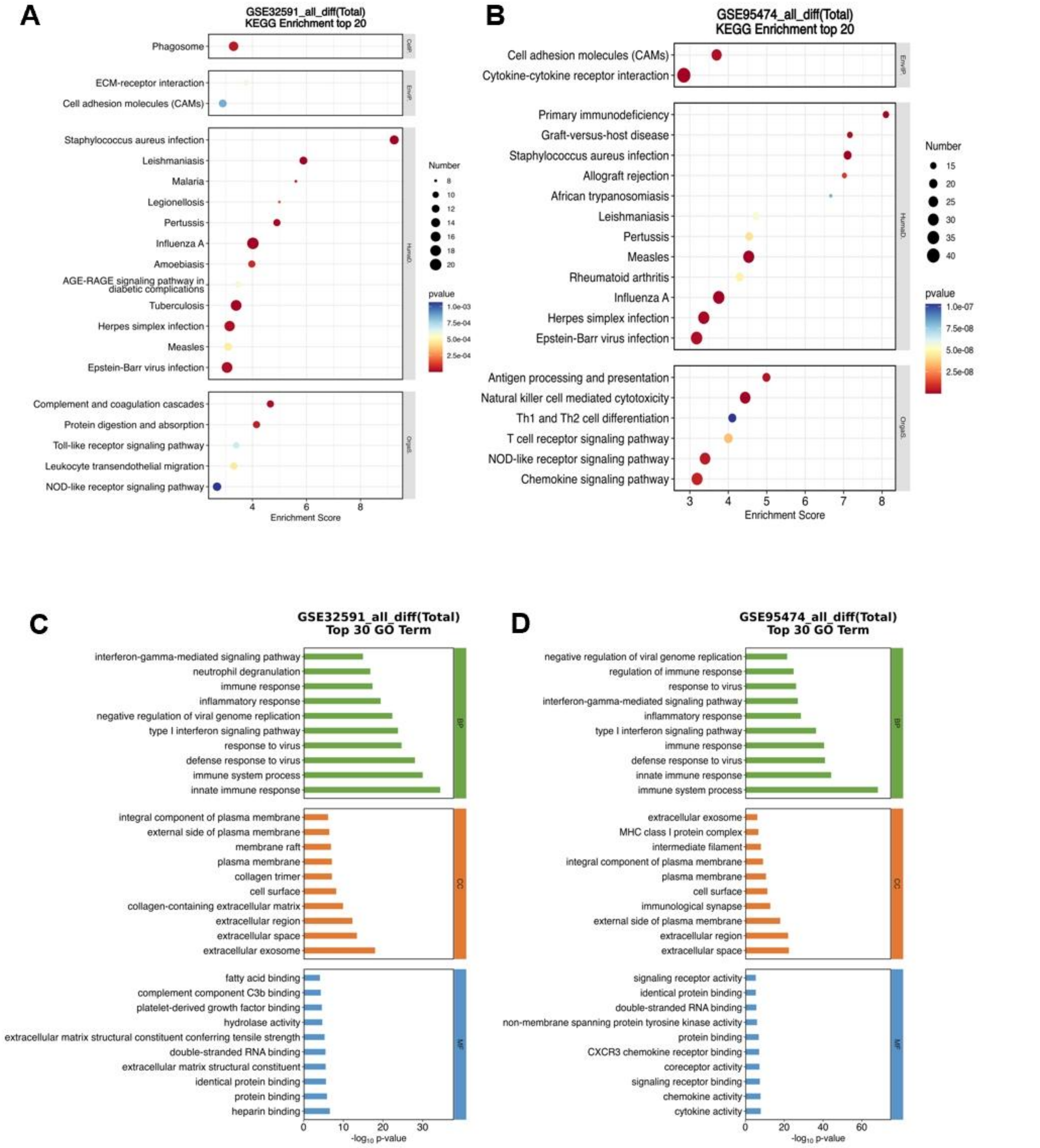

Supplementary Figure 3. Functional enrichment analyses of DEGs in LN and CLE datasets. (A,B) KEGG pathway enrichment analysis of differentially expressed genes (DEGs) in LN (A, GSE32591) and CLE (B, GSE95474). The top 20 enriched pathways are shown. Dot size indicates the number of enriched genes, and color reflects adjusted p-values. (C,D) GO enrichment analysis of DEGs in LN (C, GSE32591) and CLE (D, GSE95474). The top 30 enriched GO terms are presented, grouped into biological process (green), cellular component (orange), and molecular function (blue).

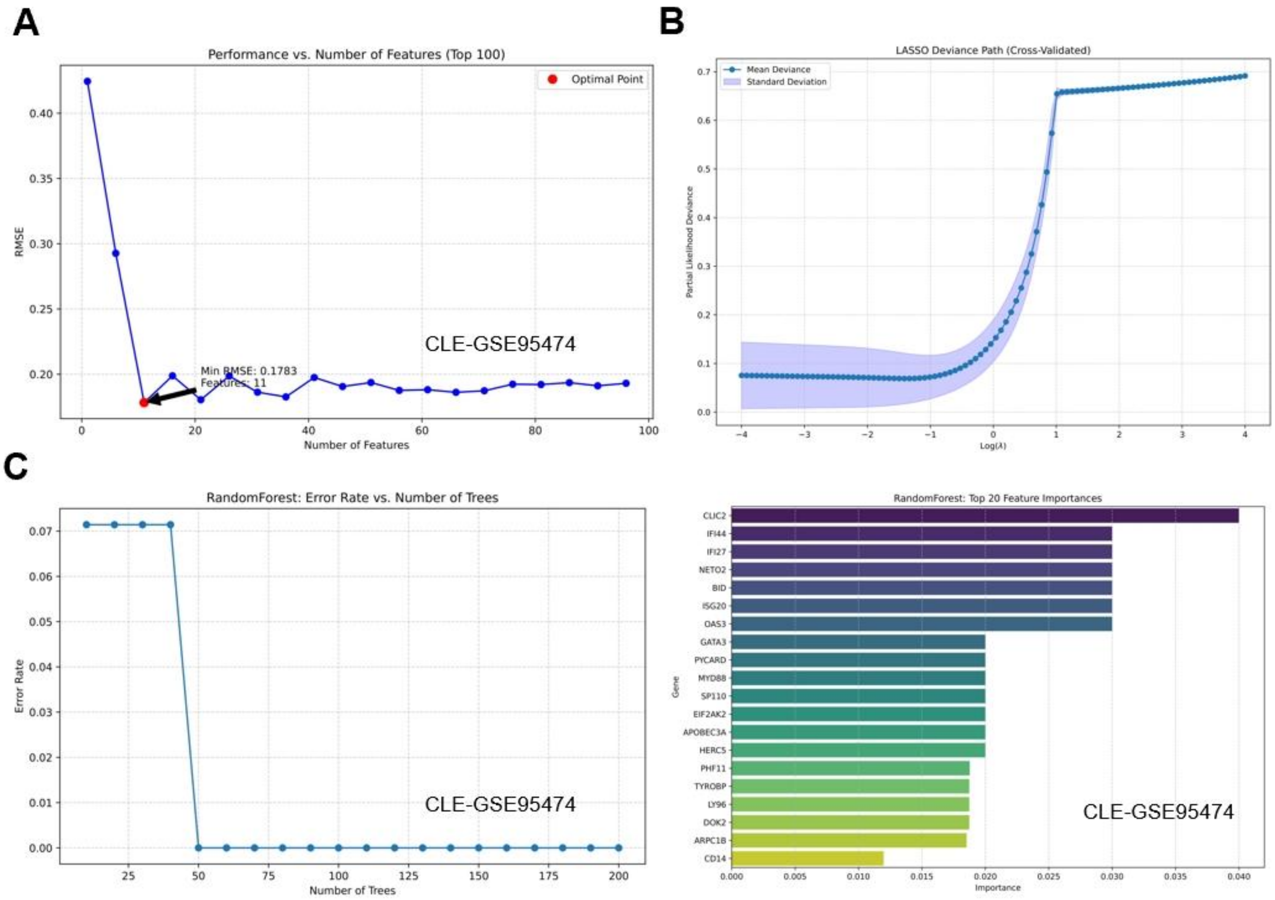

Supplementary Figure 4. Construction and evaluation of machine learning models for hub gene selection for CLE. (A) Support vector machine (SVM) recursive feature elimination (RFE) curves showing the optimal number of features for CLE (GSE95474). (B) Least absolute shrinkage and selection operator (LASSO) regression analysis in CLE. (C) Random forest (RF) analysis of CLE.

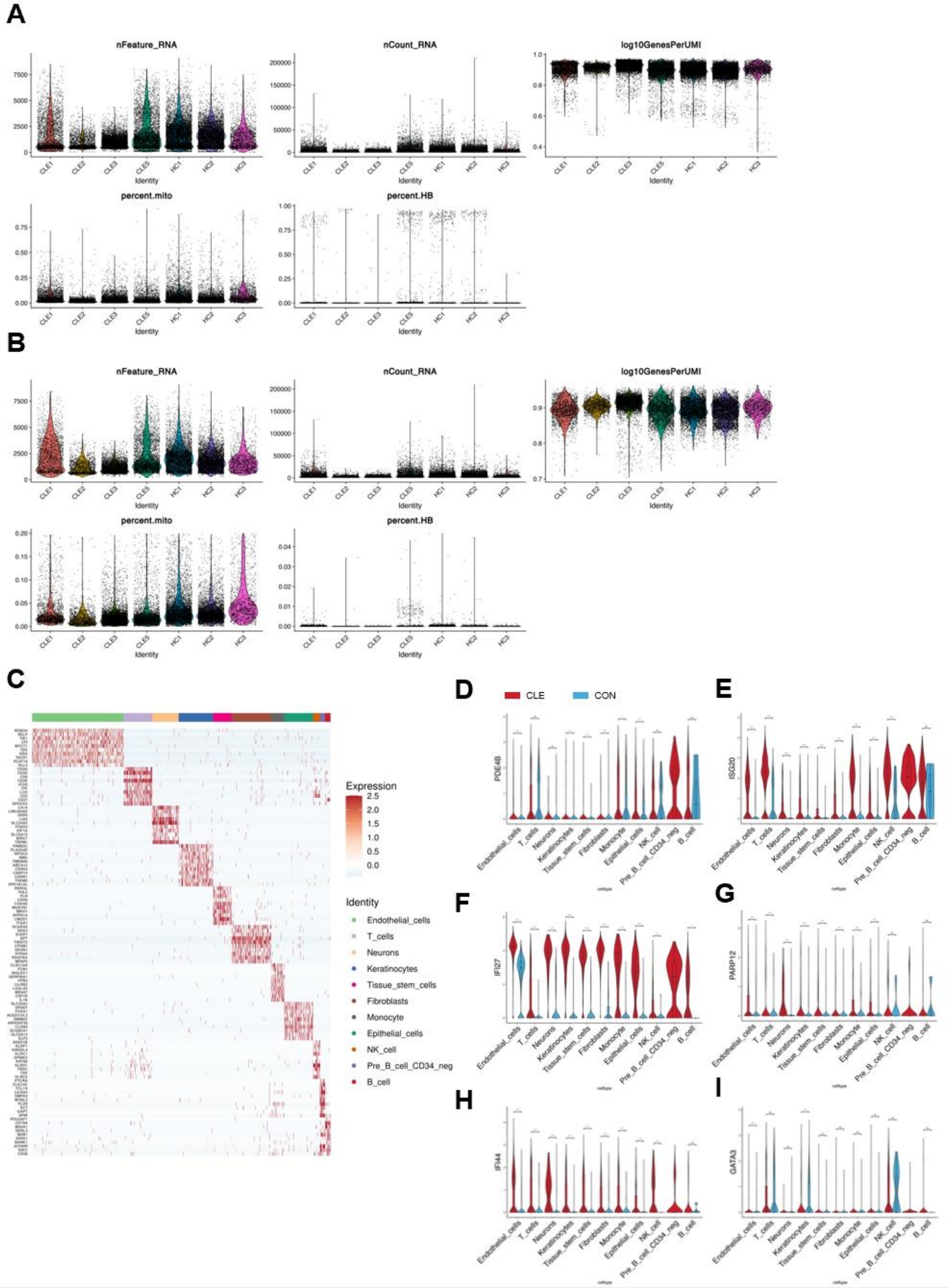

Supplementary Figure 5. Quality control and identification of skin cell populations in Single-Cell Data of CLE. (A,B) Quality control of peripheral blood mononuclear cells (PBMCs) from CLE patients and healthy controls, showing the dataset before (A) and after (B) the exclusion of low-quality cells during the initial filtering process. (C) Heatmap illustrating the expression of canonical marker genes across identified cell populations. (D-I) Differential analysis of hub genes based on single-cell data. Data are presented as mean ± SEM. *$p < 0.05$, **$p < 0.01$, ***$p < 0.001$, ****$p < 0.0001$; ns, not significant.

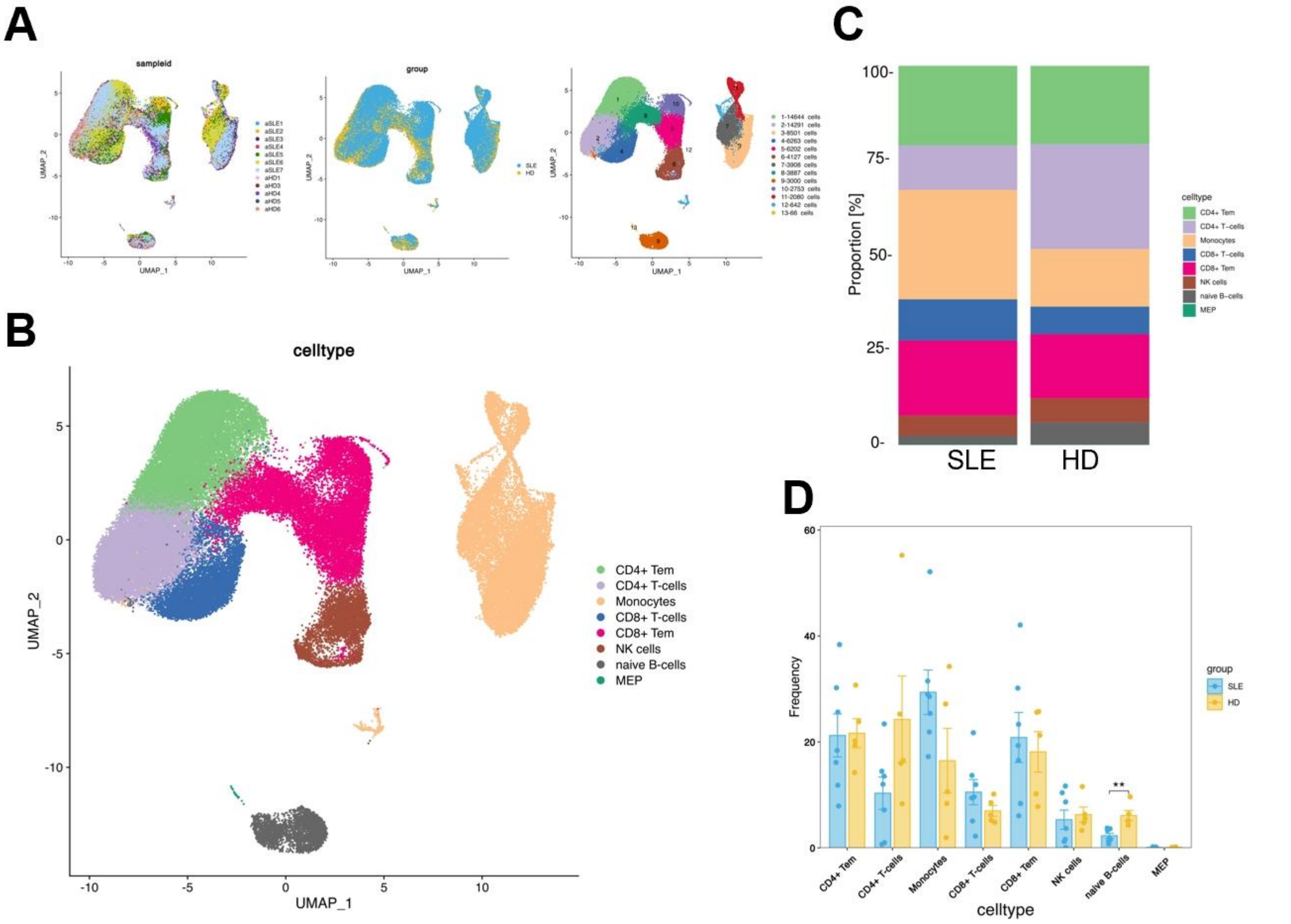

Supplementary Figure 6. PBMC Single-cell dataset validates key gene expression. (A) Data integration, normalization, and standardization across all samples, and Uniform Manifold Approximation and Projection (UMAP) clustering into 8 clusters. (B) UMAP plot of all single cells, annotated by major cell types with distinct color coding in SLE single-cell dataset. (C) Bar chart showing the distribution of major immune cell subsets between SLE patients and healthy donor (HD). (D) Frequency of each cell subsets in SLE and HD.

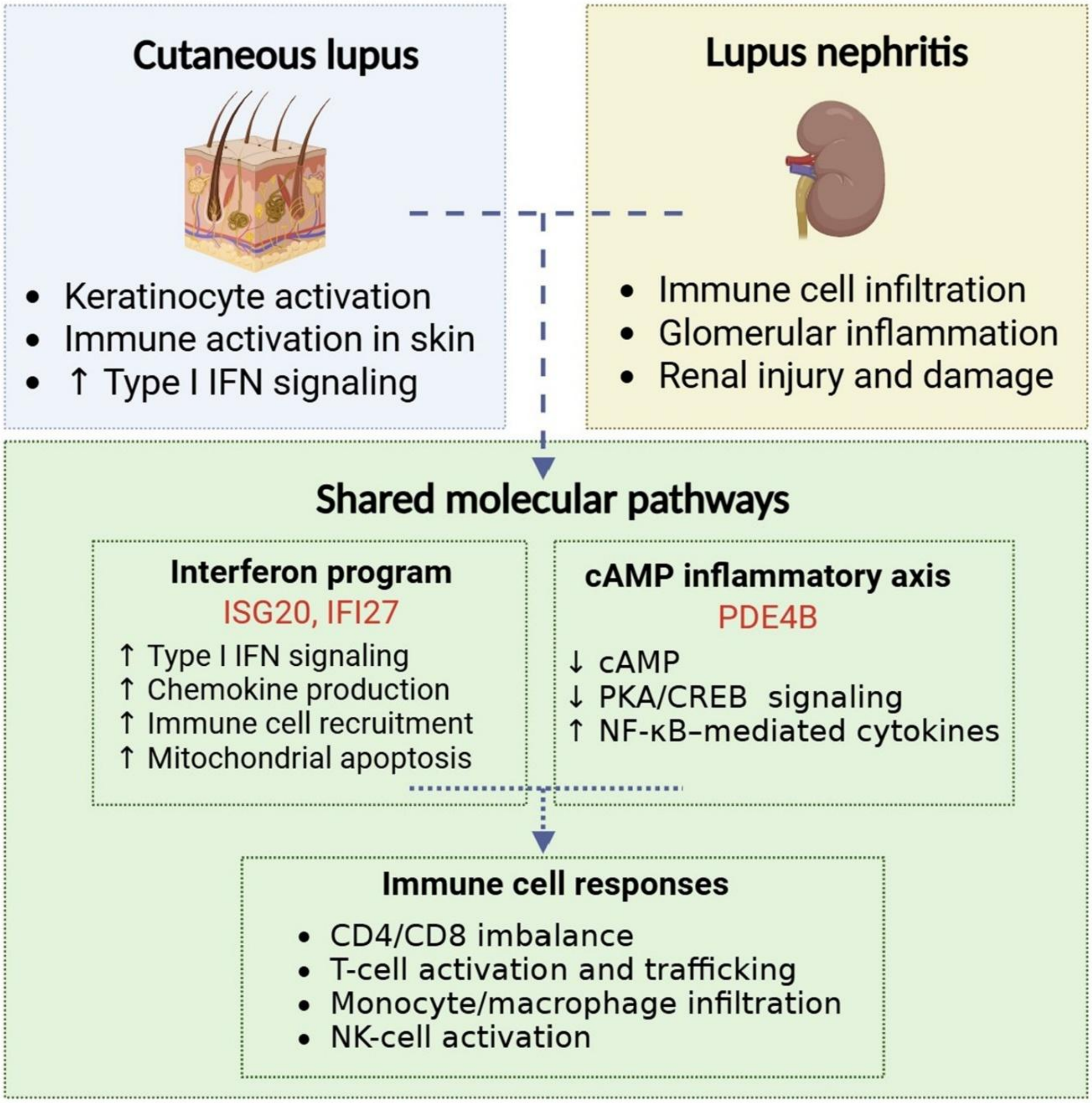

Supplementary Figure 7. Integrated mechanistic model linking cutaneous inflammation and renal involvement in lupus. In cutaneous lupus lesions, type I interferon activation triggers upregulation of interferon-stimulated genes such as ISG20 and IFI27, promoting chemokine production, immune cell recruitment, and mitochondrial stress responses. In parallel, increased PDE4B expression reduces intracellular cAMP, attenuates PKA/CREB activity, and enhances NF-κB–dependent inflammatory signaling, thereby lowering the threshold for immune activation. These molecular perturbations converge to drive dysregulated T-cell and myeloid-cell responses, facilitating trafficking of effector lymphocytes and inflammatory monocytes/macrophages from the skin into the circulation and ultimately into the kidney. The resulting immune infiltration contributes to glomerular inflammation and tissue injury, offering a mechanistic basis for the shared transcriptomic signatures observed in CLE and LN.

2 Supplementary Tables

Supplementary Table 1. GSE32591 dataset.

Supplementary Table 2. GSE95474 dataset.

Supplementary Table 3. The shared genes of DEG and WGCNA.

Supplementary Table 4. ML feature weights by model.
